# Global ubiquitinome analysis reveals the role of E3 ubiquitin ligase FaBRIZ in strawberry fruit ripening

**DOI:** 10.1093/jxb/erac400

**Published:** 2022-10-10

**Authors:** Yuying Wang, Lingxi Kong, Weihao Wang, Guozheng Qin

**Affiliations:** Key Laboratory of Plant Resources, Institute of Botany, Chinese Academy of Sciences, Beijing 100093, China; China National Botanical Garden, Beijing 100093, China; Key Laboratory of Plant Resources, Institute of Botany, Chinese Academy of Sciences, Beijing 100093, China; University of Chinese Academy of Sciences, Beijing 100049, China; Key Laboratory of Plant Resources, Institute of Botany, Chinese Academy of Sciences, Beijing 100093, China; China National Botanical Garden, Beijing 100093, China; Key Laboratory of Plant Resources, Institute of Botany, Chinese Academy of Sciences, Beijing 100093, China; China National Botanical Garden, Beijing 100093, China; University of Chinese Academy of Sciences, Beijing 100049, China; University of Birmingham, UK

**Keywords:** Fruit ripening, protein post-translational modification, strawberry, ubiquitination, ubiquitinome, ubiquitin-proteasome system (UPS)

## Abstract

Ubiquitination is an important post-translational modification that mediates protein degradation in eukaryotic cells, participating in multiple biological processes. However, the profiling of protein ubiquitination and the function of this crucial modification in fruit ripening remain largely unknown. In this study, we found that suppression of proteasome by the inhibitor MG132 retarded strawberry fruit ripening. Using K-ɛ-GG antibody enrichment combined with high-resolution mass spectrometry, we performed a comprehensive ubiquitinome analysis in strawberry fruit. We identified 2947 ubiquitination sites for 2878 peptides within 1487 proteins, which are involved in a variety of cellular functions. The lysine at position 48 (K48)-linked poly-ubiquitin chains appeared to be the most prevalent type of modification among the identified ubiquitinated proteins. A large number of ubiquitination sites exhibited altered ubiquitination levels after proteasome inhibition, including those within ripening-related proteins associated with sugar and acid metabolism, cell wall metabolism, anthocyanin synthesis, and ABA biosynthesis and signalling. We further demonstrated that FaBRIZ, a RING-type E3 ligase, functions as a negative regulator of ripening in strawberry fruit. Our findings highlight the critical regulatory roles of protein ubiquitination in fruit ripening. The ubiquitinome data provide a basis for further exploration of the function of ubiquitination on specific proteins.

## Teaser

Using ubiquitinome analysis we unravel several hundreds of ubiquitination sites on proteins in strawberry fruit, and identify a RING-type E3 ligase, FaBRIZ, which functions as an important negative regulator of ripening.

## Introduction

Ubiquitination is an important post-translational protein modification process, which is catalysed by a cascade of enzymic reactions involving ubiquitin-activating enzyme (E1), ubiquitin-conjugating enzyme (E2), and ubiquitin ligase (E3), resulting in the marking of substrate proteins with different types of ubiquitin (Ub) tags such as mono-ubiquitin, multi-ubiquitin and poly-ubiquitin chains (Callis, 2014). Because ubiquitin itself contains seven lysines (K6, K11, K27, K29, K33, K48, and K63) that are all capable of being ubiquitinated by another ubiquitin molecule, the types of poly-ubiquitin chains attached to the substrate target are very complex (Xu *et al.*, 2009). These diverse types of ubiquitination commit different fates to ubiquitinated substrates. Generally, a canonical K48-linked poly-ubiquitin chain is believed to be the principal signal for protein transferring to 26S proteasome for degradation (Xu *et al.*, 2009; Callis, 2014). As a major contributor to cellular protein degradation, the ubiquitin-proteasome system (UPS) can selectively degrade substrate proteins through specific recognition by E3s, ubiquitin receptors, deubiquitinating enzymes as well as proteasome subunits (Bard *et al.*, 2018). UPS-mediated regulation of protein stability has been demonstrated to play crucial roles in multiple biological processes in plants, such as photosynthesis, photomorphogenesis, phytohormone signalling, pathogen resistance, and stress responses (Callis, 2014; Sharma *et al.*, 2016).

Fleshy fruits are unique organs for flowering plants, which not only contribute towards seed protection and disposal, but also serve as important components of diet for humans and animals (Giovannoni, 2004). As a complex developmental process involving numerous physiological and biochemical reactions, fruit ripening is generally hallmarked by dramatic changes, such as colour alteration, loss of firmness, and accumulation of flavour and aroma compounds (Giovannoni, 2004; Liu *et al.*, 2020). Multiple environmental and internal factors have been demonstrated to control fruit ripening, including light, temperature, phytohormones, transcription factors and other developmental genes (Giovannoni, 2004; Matas *et al.*, 2009; T. Chen *et al.*, 2020; Qiao *et al.*, 2021). The phytohormone ethylene plays a critical role in the ripening of climacteric fruits (e. g. tomato, apple, and banana), while abscisic acid (ABA) is considered to contribute to the ripening of non-climacteric fruits (e.g. strawberry, grape, and citrus) (Jia *et al.*, 2011; Li *et al.*, 2021). A number of transcription factors act upstream of ethylene or ABA signalling and participate in the regulation of fruit ripening (Giovannoni *et al.*, 1995; Vrebalov *et al.*, 2002; Manning *et al.*, 2006; Seymour *et al.*, 2011; Daminato *et al.*, 2013; Wang *et al.*, 2021). Although prior studies largely focused on the molecular mechanism of fruit ripening at the transcriptional level, recent studies have shown that epigenetic and post-transcriptional regulation play critical roles in fruit ripening, such as DNA and RNA methylation (Lang *et al.*, 2017; Zhou *et al.*, 2021), histone post-translational modification (Lü *et al.*, 2018; Hu *et al.*, 2021), and non-coding RNA (Zhu *et al.*, 2015; Li *et al.*, 2018). In contrast, the roles of the UPS-mediated protein degradation in fruit ripening are still poorly understood. A comprehensive understanding of the molecular link between UPS and fruit ripening will facilitate genetic engineering for the control of fruit ripening and the improvement of fruit quality.

To date, several studies have revealed the function of UPS components in the regulation of fruit ripening. The E3 ligases MaXB3 and MaEBF1 in banana (Shan *et al.*, 2020; Song *et al.*, 2022), Sl-EBF1/EBF2 in tomato (Yang *et al.*, 2010), and VIPUB38 in grape (Yu *et al.*, 2021), were demonstrated to play important roles in fruit ripening by targeting the key proteins involved in ethylene or ABA synthesis and signalling. Meanwhile, the E3 ligases MaBRG2/3 in banana (Yang *et al.*, 2022) and MdPUB29 in apple (Hu *et al.*, 2019) were reported to regulate fruit ripening via controlling the stability of ripening-related transcription factors MaMYB4 and MdbHLH3, respectively. Other E3 ligases such as MdPUB24 in apple (Wei *et al.*, 2021) and SP1 and CUL4-DDB1-E3 ligase complex in tomato (Tang *et al.*, 2016; Ling *et al.*, 2021) were shown to participate in the development of chloroplasts and chromoplasts during fruit ripening; they could mediate the ubiquitination and subsequent proteasome-dependent degradation for the chloroplast protein import-related TOC, the chlorophyll degradation-related transcription factor MdBEL7, and the plastid level and pigment accumulation-related transcription factor GLK2, respectively.

Our previous study indicated that the E2 ubiquitin-conjugating enzyme SlUBC32 functions in the ripening of tomato fruit, and one of SlUBC32 interactors, the plastid protein sensing E3 ligase (PPSR1), could modulate the steady-state level of PSY1 protein, the main rate-limiting enzyme in the carotenoid biosynthetic pathway, thereby regulating carotenoid biosynthesis (Y. Wang *et al.*, 2014; P. Wang *et al*., 2020). Overall, these studies focused on a single regulatory event occurring between a certain E3 and its substrates, and so far, only a limited number of ubiquitinated proteins have been characterized to be regulated by UPS. It is necessary to carry out a large-scale identification of the ubiquitinated proteins and the corresponding ubiquitination sites in fruit to obtain a global understanding of UPS-mediated regulation of fruit ripening.

Recently, a state-of-the-art technique, which couples high-resolution mass spectrometry (MS) with a commercialized antibody that specifically recognizes the lysine residues modified by diglycine remnant (K-ɛ-GG), an adduct left at sites of ubiquitination after trypsin digestion, has achieved significant improvement in the detection of ubiquitinated proteins and the corresponding ubiquitination sites (Xu *et al.*, 2010). This enables researchers to identify a large number of ubiquitinated proteins in different eukaryotic species including crop plants (Zhang *et al.*, 2017; Wang *et al.*, 2019; Zhu *et al.*, 2020). Strawberry is not only a commonly consumed fresh fruit, but also a model system for ripening analysis of non-climacteric fruits (Giovannoni, 2004; T. Chen *et al.*, 2020). Although advances have been made in the molecular mechanism of strawberry fruit ripening, little is known about the regulatory effects of ubiquitination in this process (Li *et al.*, 2022).

In the present study, we found that a proteasome inhibitor MG132 retarded strawberry fruit ripening. We then performed a comprehensive proteomic analysis of ubiquitinated proteins in strawberry fruits treated with or without MG132, by using nano-HPLC-MS/MS in combination with the K-ɛ-GG antibody immunoaffinity technique. A number of ubiquitinated proteins and the ubiquitination sites were identified, and we revealed that MG132-caused ripening delaying may be correlated with the perturbance of turnover of ripening-related proteins, especially for some key ripening-related enzymes. Furthermore, we paid particular attention to several RING-type E3 ligases and demonstrated that one of these RING-type E3 ligases, FaBRIZ, negatively regulates strawberry fruit ripening. Our study provides helpful information for future exploration of functions of ubiquitinated proteins in fruit ripening, and highlights the regulatory effects of UPS in the ripening of strawberry fruit.

## Materials and methods

### Fruit material and treatment

Octoploid strawberry plants (*Fragaria*×*ananassa* ‘Benihoppe’) were grown in a plastic greenhouse under the standard culture conditions with regular fertilizers. Fruits were harvested at four different developmental stages: large green (LG), white fruit (Wt), initial red (IR), and full red (FR), approximately corresponding to 14, 21, 23, and 28 d post-anthesis, respectively. For MG132 inhibitor treatment, fruits at the large green stage were harvested and evenly injected with 100 μM MG132 (Sigma, USA) dissolved in 1% (v/v) DMSO using a syringe, until the whole fruit became hydrophanous. The negative control fruits were injected with 1% (v/v) DMSO solution. MG132-treated and control fruits were immediately collected 4 h after injection or kept at 25 °C and 80% relative humidity. The experiment was performed with more than three independent biological replicates, with each treatment containing at least 20 fruits. The collected fruits were sampled, frozen, and stored at –80 °C for further analysis. *Nicotiana benthamiana* plants were cultivated in a growth room at 22 °C under a 16 h light/8 h dark photoperiod.

### Protein extraction and western blotting

Protein extraction from strawberry fruits were performed using a phenol extraction method, as described previously in tomato fruits by Wang *et al.* (2020). Briefly, 5 g of frozen fruit tissue were finely powdered in liquid nitrogen and then thoroughly homogenized in 15 ml of extraction buffer (700 mM sucrose, 100 mM KCl, 500 mM Tris-HCl, pH 7.8, 500 mM EDTA, 1 mM PMSF, 2% w/v β-mercaptoethanol, and 1% w/v PVPP). After mixing with an equal volume of Tris-HCl saturated phenol (pH 7.5), the homogenate was vigorously vortexed, and then centrifuged at 20 000×*g* for 20 min. The upper phenol phase was collected and extracted twice with the extraction buffer. Protein was precipitated from the phenol phase by the addition of five volumes of saturated ammonium acetate in methanol overnight at –20 °C. The protein pellet was then washed twice with ice-cold methanol and ice-cold acetone, air-dried and stored at –80 °C until use.

For immunoblot analysis, the extracted fruit proteins were resuspended in lysis buffer (20 mM HEPES, pH 8.0, and 8 M urea) and protein concentrations were measured using Bradford reagent. Aliquots of protein (15 μg) were separated by 10% SDS-PAGE and then transferred to an Immobilon-P PVDF membrane (Millipore, IPVH00010, USA) using a semi-dry transfer unit (Amersham, TE77, USA). The membranes were blocked for 2 h at 25 °C with 1% BSA in TBST buffer and then subjected to immunoblotting with anti-ubiquitin antibody (P4D1; Santa Cruz Biotechnology, USA) for 1 h. After washing three times with TBST buffer, the corresponding secondary antibody conjugated to horseradish peroxidase (Abmart, China) was added. The immunoreactive bands were visualized by using a chemiluminescence detection kit (Mei5bio, MF074-01, China). Equal loadings were confirmed with an anti-actin antibody (Abmart).

### Fruit quality assessment

Fruit soluble solid content (SSC) and titratable acid (TA) were measured using Abbe refractometer (10 481 S/N, LEICA, USA) and digital acidity meter (gmk-835, g-won HITECH Co., Ltd., Korea), respectively. Total sugar content was determined using the 3,5-dinitrosalicylic acid (DNS) method with a total sugar content detection kit (Solarbio, China). Sugar content was expressed as mg glucose equivalent g^−1^ fresh weight. Total anthocyanins were measured as described by Solfanelli *et al.* (2006). In brief, 1 g of fruits was homogenized in a 3 ml mixture of 1% HCl in methanol (v/v) overnight at 25 °C. After addition of 3 ml of chloroform, the extract was centrifuged to remove debris and to separate the organic phase. The upper supernatant was collected to measure the optical density at 535 nm, with 1% methanol-HCL solution (v/v) serving as the blank control. The content of anthocyanins was expressed as A_535_ g^−1^ fresh weight.

### Ubiquitinated peptide enrichment and identification

Ubiquitinated peptide enrichment was performed using the PTMScan Ubiquitin Remnant Motif (K-ɛ-GG) kit (Cell Signaling Technology, USA) as described previously (Wang *et al.*, 2020). Briefly, proteins were extracted from MG132-treated and control fruits followed by solubilization in lysis buffer (20 mM HEPES, pH 8.0, and 8 M urea) as described above. Approximately 10 mg of the isolated proteins were reduced with 10 mM dithiothreitol at 60 °C for 30 min, and alkylated with 50 mM iodoacetamide at 25 °C in the dark for 30 min. After dilution with HEPES buffer to a final concentration of 1 M urea, the protein solution was digested with 10 ng μl^−1^ trypsin at 37 °C overnight. Following desalting on a Sep-Pak C18 column (Waters, USA), the tryptic peptides were lyophilized under vacuum. The dried peptides were resuspended in immunoprecipitation purification (IAP) buffer (Cell Signaling Technology), and then incubated with anti-K-ɛ-GG motif antibody beads (Cell Signaling Technology) for 2 h at 4 °C. The beads were collected and washed three times with IAP buffer. After elution from the beads with 0.15% trifluoroacetic acid (TFA), the ubiquitinated peptides were desalted with C18 Stage Tips (Thermo Scientific), and subjected to LC-MS/MS analysis.

MS analysis was performed with an Easy nLC-1200 system (Thermo Fisher Scientific, Waltham, MA, USA) connected with a Thermo Orbitrap Fusion Lumos mass spectrometer (Thermo Fisher Scientific). The ubiquitinated peptides were separated on a manually filled reverse phase C18 column (150 μm×25 cm, 1.9 μm particle size, 120 Å pore diameter; Dr.Maisch GmbH Inc., Germany). The mobile phase A was 0.1% formic acid in water, and the mobile phase B was 0.1% formic acid and 20% water in acetonitrile. Peptides were eluted using a linear gradient of 7–90% mobile phase B over 90 min at a flow rate of 600 nl min^-1^. The gradient used was: 7–28% B for 65 min, 28–40% B for 15 min, 40–90% B for 1 min, and 90% B for 9 min. Thermo Orbitrap Fusion Lumos mass spectrometer was used for sample analysis with the following parameters: in MS1, the resolution was set to 120 K, the scan range was 350–1550 m/z, the automatic gain control (AGC) targets were 4e5, and the charge state was set to 2–7. In MS2, the normalized collision energy was set to 32%. Ions were broken by higher collision dissociation and then analysed by orbitrap with AGC targets set at 5E4.

Protein identification and label-free quantification (LFQ) was carried out using Proteome Discoverer software (version 2.4) with Sequest search engine. Database search was performed against the strawberry protein database (*Fragaria*×*ananassa* Camarosa Genome Assembly v1.0.a1; http://www.rosaceae.org/species/fragaria_x_ananassa/genome_v1.0.a1) with the following parameters: the precursor mass tolerance was 20 ppm with a fragment mass tolerance of 0.05 Da; trypsin was set as the specific enzyme and maximum number of missed cleavages was set to 2; carbamidomethyl (C) was set as fixed modification with variable modifications of GlyGly (K), Oxidation (M), and Acetyl (Protein N-term) for all the software programs. The rest of the parameters were set as default. For LFQ analysis, normalization mode and scaling mode in precursor ions quantifier were set as ‘total peptide amount’; proteins with a ‘protein false discovery rate (FDR) confidence combined’ as ‘High’ and ‘Master’ as ‘IsMasterProtein’ were used for analysis. The rest of the parameters were set as default. Peptides ratios with fold change>2.0 or<0.5 (*P*<0.05, Student’s *t* test) cut-off were considered statistically signiﬁcant.

### Tandem Mass Tag (TMT) labelling and quantitative proteomic analysis

For proteome analysis, proteins from MG132-treated and control fruits were extracted and solubilized as described above. About 100 μg of proteins from each sample were reduced, alkylated, and digested following the above procedure. After drying and desalting, tryptic peptides were labelled with TMT reagents 6-lex Kit (Thermo Scientific) according to the manufacturer`s protocol. Three independent biological replicates were performed. The TMT-labelled peptides were then combined, lyophilized, and fractionated with high-pH reversed-phase chromatography on a Vanquish™ Flex UHPLC system (Thermo Fisher Scientific, Waltham, MA, USA) equipped with a Waters ACQUITY UPLC C18 column (2.1 × 100 mm) containing 1.7 μm particles. Totally, 24 fractions were collected and then combined into six pools for drying and desalting. The resultant peptides were analysed by LC-MS/MS as described above.

Protein identification and relative quantification were performed by Proteome Discoverer software (version 2.4). The mass spectra data were used to search the strawberry protein database (*Fragaria*×*ananassa* Camarosa Genome Assembly v1.0.a1; http://www.rosaceae.org/species/fragaria_x_ananassa/genome_v1.0.a1) using the following parameters: the precursor mass tolerance was 20 ppm with a fragment mass tolerance of 0.05 Da; trypsin was set as the specific enzyme and maximum number of missed cleavages was set to 2; carbamidomethyl (C) was set as fixed modification with variable modifications of six-plex TMT modifications (K and peptide N-terminal), Oxidation (M), and Acetyl (Protein N-terminal). The rest of the parameters were set as default. To determine the global FDR for peptide identification, a reverse database search strategy was applied. Only proteins identified below the 1% global FDR were utilized to calculate the meaningful cut-off value. Protein ratios with fold change>1.5 or<0.67 (*P*<0.05, Student’s *t* test) cut-off were considered statistically signiﬁcant.

### Bioinformatic analysis

For the identification of ubiquitinated-peptide sequence motifs, 20 residues surrounding the modified lysine (10 amino acids upstream and downstream of the ubiquitinated lysine) were extracted, aligned and visualized by iceLogo software (Colaert *et al.*, 2009). With the strawberry proteome as the background database, over- and under-represented amino acids were statistically calculated using a binomial test. Protein-protein interaction (PPI) networks were created using the STRING database (version, 11.0) with a confidence score>0.7, and visualized by Cytoscape software (version 3.7.2; Shannon *et al.*, 2003). Sub-cellular localization of proteins was predicted using an online tool Plant-mSubP (http://bioinfo.usu.edu/Plant-mSubP/). Gene Ontology (GO) annotation was performed with UniProt-GOA database (http://www.ebi.ac.uk/GOA/) using InterProScan software on the basis of protein sequence alignments (Jones *et al.*, 2014). GO enrichment was analysed using a two-tailed Fisher’s exact test (Perl module v.1.31; https://metacpan.org/pod/Text::NSP::Measures::2D::Fisher) through calculating the enrichment of the ubiquitinated proteins with up- or down-regulated ubiquitinated sites against all identified proteins. Only GO terms with a *P* value<0.05 were considered to be enriched. The heatmaps were generated using TBtools software (C. Chen *et al.*, 2020). Protein domains were annotated using InterProScan on the InterPro domain database (http://www.ebi.ac.uk/interpro/).

### *In vivo* ubiquitination assay

The ubiquitination assays of E3 ubiquitin ligases were performed with a transient expression system in *N. benthamiana* as described by Wang *et al.* (2020). Briefly, the coding regions of *XB3 Ortholog 3* in *Arabidopsis thaliana* (*XBAT34*), *PRC1 core component AtRING1* (*RING1*), *RING membrane-anchor 1* (*RMA*), *COP1 interaction protein 8* (*CIP8*), *Arabidopsis Tóxicos en Levadura 2* (*ATL2*), *Arabidopsis Tóxicos en Levadura 8* (*ATL8*), and *BRAP2 RING ZnF UBP domain-containing protein* (*BRIZ*) were amplified from the cDNA of strawberry fruits and inserted into the pCambia2300-MCS-HA vector, individually. The resulting constructs were separately transformed into *Agrobacterium tumefaciens* strain GV3101. After incubation at 28 °C for 24 h, the Agrobacteria were collected by centrifugation, resuspended in infiltration buffer (10 mM MES, pH 5.6, 10 mM MgCl_2_, and 100 μM acetosyringone) to a final OD_600_ of 1.0, and infiltrated into *N. benthamiana* leaves using a syringe. Following 30-42 h of culture, the agroinfiltrated leaves were treated with 50 μM MG132 or DMSO for 6 h, and then harvested. Total proteins from the leaves were extracted with 1 ml of extraction buffer (50 mM Tris-HCl, pH 7.5, 100 mM NaCl, 1 mM EDTA, 1% Triton X-100, 5% glycerol, 1 mM PMSF, and 1× protease inhibitor cocktail), centrifugated at 12 000×*g* for 20 min at 4 °C, and immunoprecipitated with 20 μl of anti-HA beads (Cell Signaling Technology, USA) at 4 °C for 2 h. The beads were collected and washed three times with extraction buffer. The proteins were eluted from the beads with 1× SDS loading buffer at 95 °C for 5 min, and then subjected to immunoblotting using anti-HA (Abmart, China) or anti-Flag (MBL Life Science, Japan) antibodies, respectively, as described above. The primers used for construction of vectors are listed in Supplementary Table S1.

### Protein degradation and stability assay

For the degradation assay of E3 ligase proteins, the cell-free degradation assays were performed as described by Wang *et al.* (2020) with some modifications. Briefly, pCambia2300-E3s-HA vectors were generated and transiently expressed in the *N. benthamiana* leaves as described above. Total proteins were extracted from *N. benthamiana* leaves with extraction buffer (25 mM Tris-HCl, pH 7.5, 10 mM NaCl, 10 mM MgCl_2_, 1 mM PMSF, and 5 mM dithiothreitol). Following addition of 1 mM ATP and 50 μM MG132 or DMSO (negative control), the protein extracts were incubated at 25 °C for 0, 1, 2, 3, and 4 h. The incubation mixtures were submitted to immunoblot analysis using anti-HA antibody (Abmart) as described above. The band intensity was quantified using ImageJ software (https://imagej.nih.gov/ij/index.html; Girish and Vijayalakshmi, 2004). Equal loadings were confirmed with an anti-HSP antibody (Beijing Protein Innovation, China).

### Preparation of recombinant proteins and *in vitro* ubiquitination assay

To generate MBP-tagged FaBRIZ (MBP-FaBRIZ) fusion protein, the full coding sequence of *FaBRIZ* was amplified from the cDNA of strawberry fruit and cloned into pETMALc-H vector (Wang *et al*., 2021). The resulting construct was transformed into *Escherichia coli* strain BL21 (DE3) competent cells and then expressed by induction using 1 mM IPTG (Isopropyl-thiogalactopyranoside, Sigma, USA). MBP-FaBRIZ was purified with affinity chromatography using amylose resin (New England Biolabs, E8021V, USA). Recombinant His-E1 (UBA1, M55604.1, wheat), His-E2 (UBCh5b, U39317.1, human), and His-Ub (UBQ14, At4g02890, Arabidopsis) were expressed in BL21 (DE3) and purified using Ni-NTA Agarose (QIAGEN, 30 210, Germany). The primers used for construction of vectors are listed in Supplementary Table S1.

The *in vitro* ubiquitination assay was performed as described previously (Wang *et al.*, 2020). Briefly, 500 ng of purified MBP-FaBRIZ fusion protein was mixed with 100 ng of E1, 200 ng of E2, and 2 μg of ubiquitin in 30 μl of reaction buffer (50 mM Tris-HCl, pH 7.5, 5 mM MgCl_2_, 2 mM ATP, and 2 mM dithiothreitol). The reactions were incubated at 30 °C for 2 h and stopped by adding 2× SDS sample buffer at 95 °C for 5 min. The reaction products were analysed by 8% SDS-PAGE followed by immunoblotting using anti-ubiquitin (P4D1, Santa Cruz Biotechnology) and anti-MBP (Beijing Protein Innovation) antibodies, as described above.

### Quantitative real-time PCR analysis (qRT–PCR)

Total RNA was isolated from strawberry fruits at four different developmental stages as described above using the plant RNA extraction kit (Magen, R4165-02, China). Genomic DNA was removed by incubation with RNase-Free DNase (Vazyme, R323-01, China) and reverse transcription of the extracted RNA were performed using the HiScript^®^ III RT SuperMix for qPCR kit (Vazyme, R323-01, China). Quantitative RT–PCR was performed with ChemQ Universal SYBR qPCR Master Mix (Vazyme, Q711-02-AA) using the StepOne Plus Real-Time PCR System (Applied Biosystems). PCR amplification was performed using the following program: 95 °C for 10 min, followed by 40 cycles of 95 °C for 15 s and 60 °C for 30 s. The cycle threshold (Ct) 2^(−ΔΔCt)^ method was applied to the relative quantification of gene expression level. Strawberry *ACTIN* and *GAPDH* was used to normalize the expression values. The primers for PCR amplifications are listed in Supplementary Table S1. Three biological replicates were conducted, with each containing three technical repeats.

### Agroinfiltration-mediated transient transformation in strawberry fruit

To generate the *XBAT34*, *RING1*, *CIP8*, *ATL2*, and *FaBRIZ* RNA interference (RNAi) plasmids, a fragment of 200~350 bp from the *XBAT34* gene (bases 215 to 504 of the full-length cDNA), *RING1* (bases 213 to 500 of the full-length cDNA), *CIP8* (bases 398 to 740 of the full-length cDNA), *ATL2* (bases 499 to 818 of the full-length cDNA), and *FaBRIZ* gene (bases 219 to 518 of the full-length cDNA) was sub-cloned into the pCR8 vector (Invitrogen, USA), individually, and restructured into the pK7GWIWGD(II) plasmid using the Gateway LR Clonase^TM^ Enzyme Mix (Invitrogen, 11 791-020). To generate the *FaBRIZ* overexpression (OE) vectors, the full-length sequence of *FaBRIZ* was amplified and ligated into the pCambia2300-HA-MCS vector. The resulting constructs were separately introduced into the *A. tumefaciens* strain GV3101, and then transiently transformed into strawberry fruit by agroinfiltration, as described previously (Hoffmann *et al.* 2006). In brief, the cultured Agrobacteria were resuspended in the infiltration buffer (10 mM MES, pH 5.6, 10 mM MgCl_2_, and 100 μM acetosyringone) to a final OD_600_ of 0.8. After keeping at 25 °C for 2 h without shaking, the *Agrobacteria* suspensions were infiltrated into the octoploid strawberry fruit at the large green stage using a 1 ml syringe. The infiltrated fruits were cultured for 5–7 d in a growth room (23 °C, 80% relative humidity, and a 16 h/8 h light/dark photoperiod). The experiment was performed with more than three independent biological replicates, and each replicate contained at least 20 fruits. The primers used for the vector constructions are listed in Supplementary Table S1.

### Accession numbers

Sequence data described in this article can be found in the GDR Genomics Network (https://www.rosaceae.org/) under the following accession numbers: maker-Fvb4-3-augustus-gene-295.45-mRNA-1 (*XBA34*), augustus_masked-Fvb5-1-processed-gene-112.18-mRNA-1 (*RING1*), maker-Fvb3-4-snap-gene-244.57-mRNA-1 (*RMA*), maker-Fvb2-4-snap-gene-253.58-mRNA-1 (*CIP8*), augustus_masked-Fvb4-3-processed-gene-134.13-mRNA-1 (*ATL2*), augustus_masked-Fvb1-1-processed-gene-227.7-mRNA-1 (*ATL8*), maker-Fvb6-2-augustus-gene-66.20-mRNA-1 (*FaBRIZ*), maker-Fvb1-2-augustus-gene-1.47-mRNA-1 (*SS*), maker-Fvb7-2-snap-gene-10.38-mRNA-1 (*CHS*), maker-Fvb6-1-augustus-gene-70.51-mRNA-1 (*PG1*), maker-Fvb1-2-augustus-gene-1.47-mRNA-1 (*NCED1*), maker-Fvb4-3-augustus-gene-99.43-mRNA-1 (*ACTIN*), and maker-Fvb4-4-augustus-gene-66.43-mRNA-1 (*GAPDH*).

## Results

### Suppression of proteasome delays strawberry fruit ripening

**Fig. 1.**
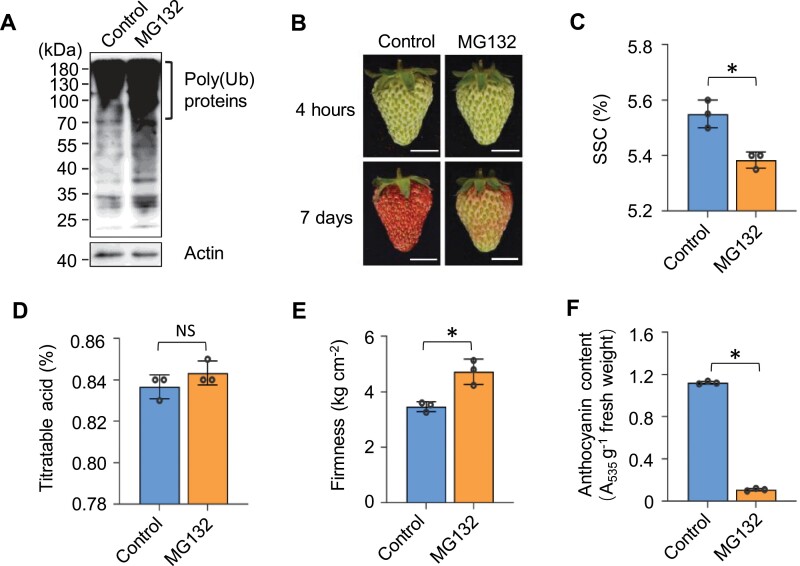
Proteasome inhibition suppresses the ripening process of strawberry fruit. Strawberry fruits at large green (LG) stage were injected with 100 μM of MG132 or DMSO solution (control). Twenty fruits were used for each treatment and the whole experiment was repeated more than three times. (A) Western blot detection of ubiquitinated proteins with anti-ubiquitin antibody (P4D1). Protein extracts were prepared from control and MG132-treated fruits after 4 h of treatment. Actin served as the loading control. (B) Ripening phenotypes of fruits after MG132 treatment for 4 h and 7 d. The representative photographs of fruits are shown. Scale bar=1 cm. (C-F) Quantification of soluble solid content (SSC) (C), titratable acid (D), fruit firmness (E), and anthocyanin content (F) in control fruits or fruits after MG132 treatment for 7 d. Data are presented as means ±SD (*n*=3). Asterisks indicate significant differences (*P<*0.05; Student’s *t* test).

To investigate whether the ubiquitin-proteasome system plays a role in fruit ripening, an inhibitor of proteasomes that inhibits the proteasome core particle, MG132, was used for treatment of octoploid cultivated strawberry fruit at the large green (LG) stage. Since inhibition of proteasome function should result in the accumulation of ubiquitinated proteins, we first examined the changes in protein ubiquitination in MG132-treated fruits using immunoblotting analysis. Total proteins extracted from control fruits and fruits treated with MG132 at 4 h were subjected to western blotting with anti-ubiquitin antibody. As shown in Fig. 1A and Supplementary Fig. S1, ubiquitinated proteins could be detected with a wide range of molecular masses (25–180 kDa), and the levels of protein ubiquitination were increased upon MG132 treatment. The abundance of ubiquitinated proteins accumulated in a MG132 dose-dependent manner, reaching saturation at 100 μM (Fig. 1A; Supplementary Fig. S1). These data confirmed that MG132 treatment is effective in blocking the proteasome. The dose of 100 μM of MG132 was used for further analysis.

Intriguingly, we noticed that MG132 treatment drastically delayed strawberry fruit ripening. After 7 d of incubation, the control fruits showed a homogenous red colour, whereas MG132-treated fruits were just beginning to change colour (Fig. 1B; Supplementary Fig. S2). Consistently, fruit quality analysis indicated that the soluble solid content (SSC) and anthocyanin concentration were lower in the MG132-treated fruits, while the fruit firmness was higher than in the controls (Fig. 1C-F). These results suggest that inhibition of proteasome function is able to suppress the fruit ripening process of strawberry. Notably, the MG132-treated fruits eventually ripened after 9 d of incubation (Supplementary Fig. S2), indicating that this suppression of fruit ripening by MG132 was not due to a toxic effect.

### Ubiquitinome profiling of strawberry fruit identifies thousands of ubiquitinated proteins

To investigate the molecular mechanism by which protein ubiquitination affects fruit ripening in strawberry, we performed ubiquitinome analysis to gain a global view of ubiquitination alteration after MG132 treatment. To exclude the influence of ripening stages on protein ubiquitination, the 4 h-treated fruits, which displayed no significant phenotypic changes, were chosen for analysis (Fig. 1B). An overview of ubiquitinome analysis workflow is shown in Supplementary Fig. S3A. Total proteins were extracted from control and MG132-treated fruits, digested with trypsin, and incubated with anti-K-ɛ-GG antibody, which specifically recognizes the lysine residues (K) modified by diglycine (diGly, GG), the remnant derived from ubiquitinated protein after tryptic digestion. The enriched diGly peptides, which represent ubiquitinated peptides, were analysed by nano-HPLC-MS/MS using a label-free quantitative method.

**Fig. 2.**
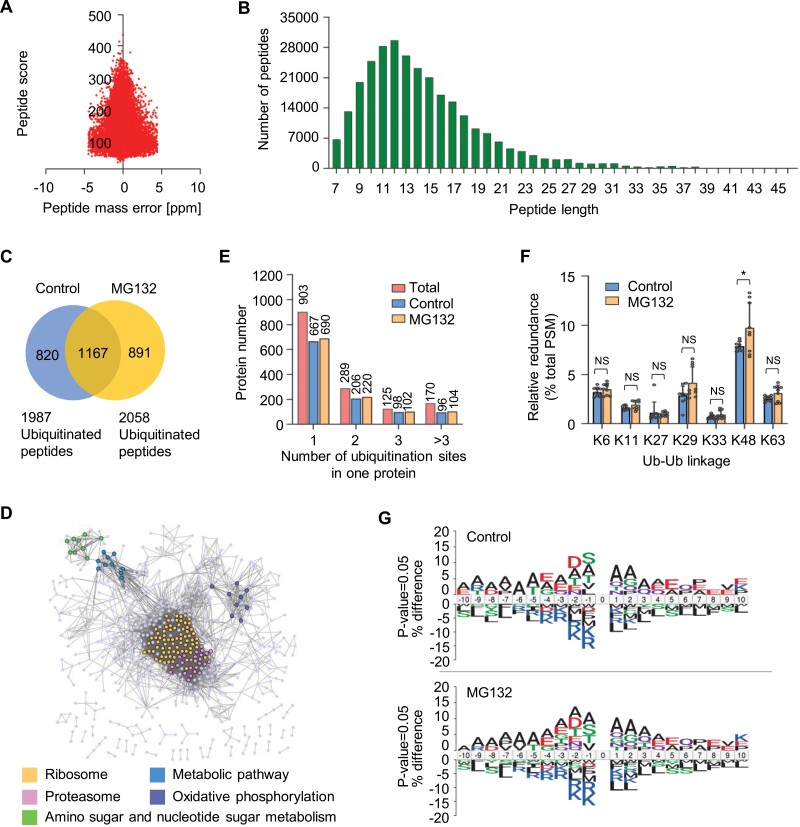
Ubiquitinome profile analysis in strawberry fruit. (A) Mass accuracy distribution of the identified peptides. (B) Length distribution of identified peptides using mass spectrometry. In total, 287 612 peptides were analysed. (C) Venn diagram showing the overlap of ubiquitinated peptides identified in strawberry fruits treated with or without 100 μM MG132 for 4 h. (D) Protein-protein interaction network of all identified ubiquitinated proteins generated by STRING v.11.0 with a high confidence score (>0.7) and visualized using Cytoscape software (v3.7.2). Bubbles indicate proteins. The related interactions between proteins are shown as grey lines. Top five enriched ubiquitinated proteins are indicated using different fill colours. (E) Numbers of the ubiquitinated proteins containing different numbers of ubiquitination sites. (F) Distribution of ubiquitin (Ub) footprints based on MS analysis. Data are presented as means ±SD (*n*=3). Asterisks indicate significant differences (*P*<0.05; Student’s *t* test). PSM, peptide spectral matches, indicative of the number of peptide spectra; NS, no significance. (G) Sequence motifs surrounding ubiquitination sites determined by iceLoGo. Ten amino acids up- and down-stream of the ubiquitinated lysine residues, respectively, were used for alignment, and the under- or over-enriched amino acid residues are shown.

We carried out three replicate ubiquitinome experiments, in which the ubiquitinated peptides were independently immunoaffinity-enriched for each treatment. The first-class mass error and the distributions of peptide length met the requirement of mass accuracy (Fig. 2A, B). Only ubiquitination sites consistently detected in at least two replicates for each treatment were regarded as high-confidence ubiquitination sites and used for subsequent analysis. In total, we identified 2947 high-confidence ubiquitination sites for 2878 peptides within 1487 proteins, of which 1987 ubiquitinated peptides within 1067 proteins, and 2058 ubiquitinated peptides within 1116 proteins were identified, respectively, in control and MG132-treated strawberry fruits (Fig. 2C; Supplementary Table S2). There were 1167 ubiquitinated peptides within 1001 proteins overlapping between the two samples (Fig. 2C; Supplementary Table S2). Gene Ontology (GO) analysis of all identified ubiquitinated proteins in control and MG132-treated fruits revealed a potential function of protein ubiquitination in multiple cellular processes (Supplementary Fig. S3B). Furthermore, based on protein-protein interaction network (PPI) analysis, five specifically enriched categories were found, including ribosome, proteasome, metabolic pathway, oxidative phosphorylation, as well as amino sugar and nucleotide sugar metabolism (Fig. 2D), indicating that protein ubiquitination may be involved in the regulation of these processes in fruits.

Most of the identified ubiquitinated proteins (>60%) contained one ubiquitination site (Fig. 2E). The numbers of ubiquitinated proteins harbouring one or more ubiquitination sites identified in MG132-treated fruits were all slightly higher than those in control fruits. There were 690, 220, 102, and 104 ubiquitinated proteins containing one, two, three, and more sites, respectively, in fruits treated with MG132, whereas 667, 206, 98, and 96 ubiquitinated proteins containing corresponding sites were identified, in the control (Fig. 2E). However, after normalization by numbers of total ubiquitinated proteins identified in control and MG132-treated samples, the distribution of ubiquitinated proteins harbouring various number of ubiquitination sites showed no difference between the two samples, likely reflecting no bias for MG132 on ubiquitination sites.

All seven lysine (K) residues in ubiquitin (Ub) contribute to the assembly of polyubiquitin chains (poly-Ub). Thus, we evaluated the type of poly-Ub linkages in identified ubiquitinated proteins through counting the peptide spectral matches (PSMs) of Ub peptides with different K-diGly sites in MS. In total, we identified 21 diGly-modified Ub peptides (totally 6644 PSMs), which mapped to all seven lysine residues in Ub (Supplementary Table S2). A similar linkage preference of K48> K29> K6> K63> K11> K27> K33 was generated for both control and MG132-treated samples (Fig. 2F). As expected, K48 linkage was significantly increased (*P*<0.05, Student’s *t* test) after MG132 treatment, while no marked changes were found for other types of linkages (Fig. 2F). This indicated that substrate modified with K48 chains might therefore be targeted to the proteasome, which is in general consistent with the observation in Arabidopsis (Kim *et al.*, 2013), showing that K48, K63, and K11 linkage were dramatically enriched after MG132 treatment, compared with other linkages.

To characterize the sequence properties of ubiquitination sites identified in strawberry fruits, we performed amino acid preference analysis using the iceLogo program. The serine (S) and aspartic acid (D) upstream of the ubiquitinated lysine (K) residues were notably over-represented in the control fruits, whereas there was a subtle enrichment for two consecutive alanines (‘AA’) upstream of the ubiquitinated K in the fruits after MG132 treatment (Fig. 2G). Both samples had preference of ‘AA’ downstream of the ubiquitinated K and showed no bias of arginine (R), lysine (K), or leucine (L) near the ubiquitination sites (Fig. 2G). Indeed, the ubiquitinated lysine K following A residue has been previously described to be a sequence feature for ubiquitination in wheat, maize, and rice (Zhang *et al.*, 2017; Wang *et al.*, 2019; Zhu *et al.*, 2020), but not in petunia, *Paeonia*, and tobacco (Guo *et al.*, 2017; Gu *et al.*, 2019; Zhan *et al.*, 2020), in which glutamic acid (E) was found upstream of the ubiquitinated K. These results suggest that the preference of ubiquitination sites exhibit species specificity.

### Quantitative analysis reveals the reprogramming of ubiquitinome upon proteasome inhibition in strawberry fruit

**Fig. 3.**
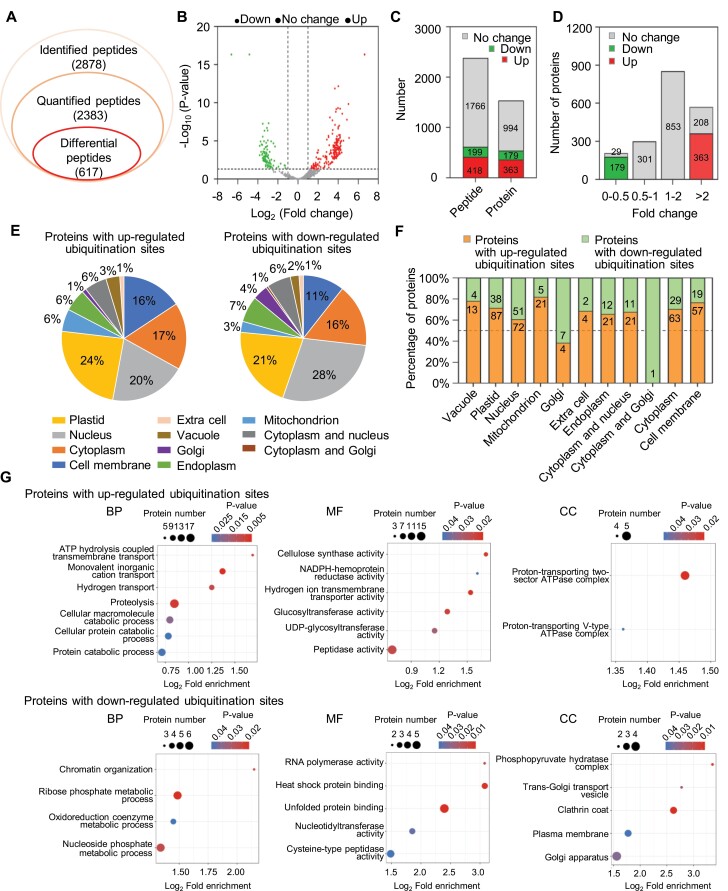
Quantitative ubiquitinome analysis upon proteasome inhibition in strawberry fruit. (A) Summary of the total identified ubiquitinated peptides, quantified ubiquitinated peptides, and differentially regulated ubiquitinated peptides. (B) Volcano plot showing the quantified ubiquitinated peptides in strawberry fruits after MG132 treatment, compared with the control. Ubiquitinated peptides with significantly higher and lower ubiquitination levels are highlighted in red and green, respectively (fold change>2.0 or<0.5; *P* value<0.05, Student’s *t* test). (C) Number of up- and down-regulated ubiquitinated peptides as well as ubiquitinated proteins corresponding to the ubiquitinated peptides. (D) Fold change distribution of proteins with differentially regulated ubiquitination sites. (E) Sub-cellular localization chart of proteins with up- or down-regulated ubiquitination sites. (F) Distribution of ubiquitinated proteins among different organelles. The number of ubiquitinated proteins assigned to each group is indicated. (G) GO enrichment analysis for ubiquitinated proteins with up- or down-regulated ubiquitination sites. The size of the bubbles indicates different numbers of proteins, and the colour represents the q value of the pathway enrichment. BP, biological process; MF, molecular function; CC, cellular components.

Next, we quantitatively analysed the changes in the ubiquitinome of strawberry fruit after MG132 treatment. Of the 2878 ubiquitinated peptides containing 2947 ubiquitination sites, 2383 peptides were quantified accurately (Fig. 3A; Supplementary Table S2). Across these quantified ubiquitinated peptides, 617 peptides were identified with differential abundance upon MG132 treatment (fold change>2.0 or<0.5, *P* value<0.05). Among them, 418 ubiquitinated peptides within 363 proteins exhibited higher abundance, and 199 within 179 proteins displayed lower abundance after MG132 treatment (Fig. 3B-D; Supplementary Table S3). These data confirmed a global increase in protein ubiquitination levels in strawberry fruit after MG132 treatment.

Since protein ubiquitination has been reported to influence protein abundance, we performed a quantitative proteomic analysis of total protein in control and MG132-treated strawberry fruits to evaluate whether there is a potential correlation between protein ubiquitination and protein abundance. A total of 8725 proteins were identified from control and MG132-treated strawberry fruits, of which only 885 proteins were detected in our ubiquitinome analysis (Supplementary Fig. S4A, B; Supplementary Table S4). The poor overlap reflects that many ubiquitinated proteins may be present in low abundance or in sub-cellular organelles, resulting in them not being amenable to be measured in the whole proteome (Anania *et al.*, 2014). Interestingly, most (>99%) proteins identified in the quantitative proteomic analysis, including the overlapping proteins identified in ubiquitinome analysis, showed no significant changes in abundance after MG132 treatment (fold change>1.5 or<0.67; *P<*0.05; Supplementary Fig. S4C, D). This result is consistent with a previous observation in human cells showing that >97% of proteins did not change abundance when the proteasome was blocked (Porras-Yakushi *et al.*, 2021). It is possible that inhibition of the proteasome induced the unfolded protein response (UPR), causing a global inhibition of translation (Larance *et al.*, 2013), which in turn neutralizes the effect of proteasome suppression. Therefore, proteins with no significant increase in abundance are also likely to be proteasome targets (Larance *et al.*, 2013). Regardless, these data confirmed that the ubiquitination level of individual peptides was not determined by protein turnover or degradation.

To further analyse the possible function of the ubiquitinated proteins, sub-cellular structure prediction was conducted using Plant-mSubP. As shown in Fig. 3E, the identified ubiquitinated proteins with up- or down-regulated ubiquitination sites upon MG132 treatment were predominantly localized in the plastid (>21%), nucleus (>20%), cytoplasm (>16%), and cell membrane (>11%). Furthermore, the percentage of proteins with up-regulated ubiquitination sites after MG132 treatment was higher than that of proteins with down-regulated ubiquitination sites in almost all cell organelles except the Golgi apparatus (Fig. 3F), indicating that targeted protein ubiquitination by the ubiquitin-proteasome system (UPS) occurs in almost all cellular compartments.

To understand the functional difference between proteins with up-regulated and down-regulated ubiquitination sites, we performed an enrichment analysis with Gene Ontology (GO) and Kyoto Encyclopedia of Genes and Genomes (KEGG) databases. We found that proteins with up-regulated ubiquitination sites were mostly enriched in the known UPS-participated pathway, such as ‘proteolysis’, ‘protein catabolic process’, and ‘peptidase activity’ (Fig. 3G; Supplementary Fig. S5A), while proteins with down-regulated ubiquitination sites mainly occurred in ‘cysteine-type peptidase’, ‘Golgi apparatus’, and ‘plasma membrane’ (Fig. 3G; Supplementary Fig. S5B), in which ubiquitination might direct autophagic turnover or other non-proteolytic outcomes. These results indicated that multiple cellular processes were influenced after proteasome inhibition.

### The ubiquitination sites of some ripening-related proteins exhibit altered levels upon proteasome inhibition

**Fig. 4.**
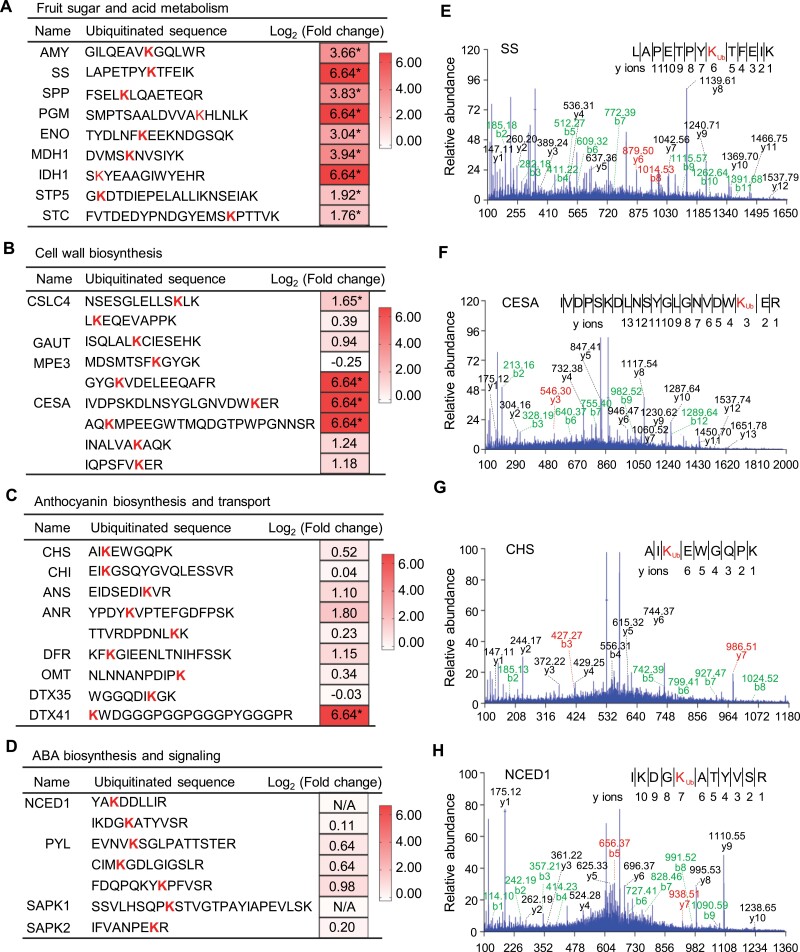
Identification of ubiquitination sites in proteins involved in strawberry fruit ripening. (A) The ubiquitinated proteins involved in sugar and acid metabolism, (B) cell wall metabolism, (C) anthocyanin biosynthesis and transport, and (D) ABA biosynthesis and signalling are listed. Heatmaps show log_2_-transformed fold changes of ubiquitination levels of the indicated ubiquitination sites after MG132 treatment. Asterisks indicate significant differences (fold change>2.0 or<0.5; *P* value<0.05, Student’s *t* test). The letter K in red indicates the ubiquitinated lysine. (E) Representative mass spectra of the ubiquitinated peptides for indicated proteins are displayed, including sucrose synthase (SS); (F) cellulose synthase A catalytic subunit 1 (CESA); (G) chalcone synthase (CHS); and (H) 9-*cis*-epoxycarotenoid dioxygenase 1 (NCED1). The y-ions and the corresponding peptide sequence are shown and the ubiquitinated lysine (K) residues are marked in red. AMY, alpha-amylase 2; ANR, anthocyanidin reductase; ANS, anthocyanidin synthase; CHI, chalcone-flavonone isomerase 3; CSLC4, xyloglucan glycosyltransferase 4; DFR, dihydroflavonol 4-reductase; 5DTX35, DETOXIFICATION 35; DTX41, DETOXIFICATION 41; ENO, enolase; GAUT, galacturonosyltransferase 3; IDH1, isocitrate dehydrogenase [NADP]; MDH1, malate dehydrogenase; MPE3, pectinesterase 3; OMT, flavonoid 3’,5’-methyltransferase; PGM, phosphoglucomutase, cytoplasmic; PYL, abscisic acid receptor PYL9; SAPK1, serine/threonine-protein kinase SAPK1; SAPK2, serine/threonine-protein kinase SAPK2; SPP, sucrose-phosphatase 1; STC, sugar carrier protein C; STP5, sugar transport protein; N/A, no quantitative values.

Given that carbohydrate metabolism, cell wall reconstruction, anthocyanin accumulation as well as ABA synthesis and signalling are key events during strawberry fruit ripening (Giovannoni, 2004), we set out to identify ubiquitinated proteins annotated to these categories. A number of proteins involved in sugar and acid metabolism were present in our ubiquitinome, ranging from sugar/acid accumulation to sugar/acid transporters (Supplementary Table S5). After MG132 treatment, the ubiquitination level of specific ubiquitination sites for majority of proteins were significantly increased (*P*<0.05, Student’s *t* test), such as alpha amylase (AMY), sucrose synthase (SS), sucrose-phosphatase 1 (SPP), phosphoglucomutase (PGM), enolase (ENO), malate dehydrogenase (MDH1), and isocitrate dehydrogenase (IDH1) (Fig. 4A, E; Supplementary Table S5), indicating them as the targeted substrates for degradation by proteasome. Notably, several sugar-related transporters, including sugar transport protein 5 (STP5) and sugar carrier protein C (STC), also contained significantly up-regulated (*P*<0.05, Student’s *t* test) ubiquitinated sites in strawberry fruits treated with MG132 (Fig. 4A; Supplementary Table S5), implying a role for proteasome-dependent protein ubiquitination in control of nutrient transport.

A set of ubiquitinated proteins involved in cell wall metabolism were present in the ubiquitinome, including xyloglucan glycosyltransferase 4-like (CSLC4), galacturonosyltransferase (GAUT), pectinesterase 3 (MPE3), and cellulose synthase A catalytic subunit 1 (CESA) (Fig. 4B, F; Supplementary Table S5). Of these ubiquitinated proteins, three proteins (CSLC4, MPE3, and CESA) contained up-regulated ubiquitinated sites (Fig. 4B; Supplementary Table S5), indicating that these enzymes are the potential targets of ubiquitin-proteasome mediated proteolysis.

A number of crucial enzymes involved in anthocyanin biosynthesis and transport were detected in our ubiquitinome, including chalcone synthase (CHS), chalcone flavonone isomerase (CHI), anthocyanidin synthase (ANS), anthocyanidin reductase (ANR), dihydroflavonol 4-reductase (DFR), flavonoid 3’,5’-methyltransferase (OMT), and detoxification efflux carriers (DTX35 and DTX41) (Fig. 4C, G; Supplementary Table S5), suggesting that anthocyanin metabolism might be substantially regulated by ubiquitination. For the ubiquitinated peptides detected, only those in DTX41, the anthocyanin transporter, were up-regulated after MG132 treatment (Fig. 4C; Supplementary Table S5). The ubiquitinated peptides in proteins involved in anthocyanin biosynthesis did not exhibit significant change. This may be due to the low abundance of these proteins at the large green stage of strawberry fruit ripening, that we used for ubiquitinome analysis.

Several key components in ABA biosynthesis and signalling pathway were identified in the ubiquitinome, including 9-*cis*-epoxycarotenoid dioxygenase 1 (NCED1), ABA receptor PYL9-like (PYL), and ABA signalling-related serine/threonine-protein kinases (SAPK1 and SAPK2) ( Fig. 4D, H; Supplementary Table S5), suggesting the potential regulatory effects of protein ubiquitination on ABA pathway in strawberry fruit. However, all the ubiquitinated peptides within proteins in the ABA pathway displayed no significant change in abundance after MG132 treatment (Fig. 4D; Supplementary Table S5). This is most likely due to the low abundance of these proteins at the ripening stage (i.e. large green stage) that we sampled for ubiquitinome analysis, which may compromise the effect of MG132.

### E3 ubiquitin ligases exhibit altered levels of ubiquitination sites after proteasome suppression

In our ubiquitinome analysis, more than 180 UPS components, representing one of the most enriched function groups, were identified, including proteasome subunits as well as enzymes involved in ubiquitination or deubiquitination (Supplementary Table S6). Among them, about 18% contained up-regulated ubiquitination sites after MG132 treatment (Supplementary Table S6), suggesting that UPS may regulate its own components through ubiquitination, which may in turn influence stability of the target proteins.

**Fig. 5.**
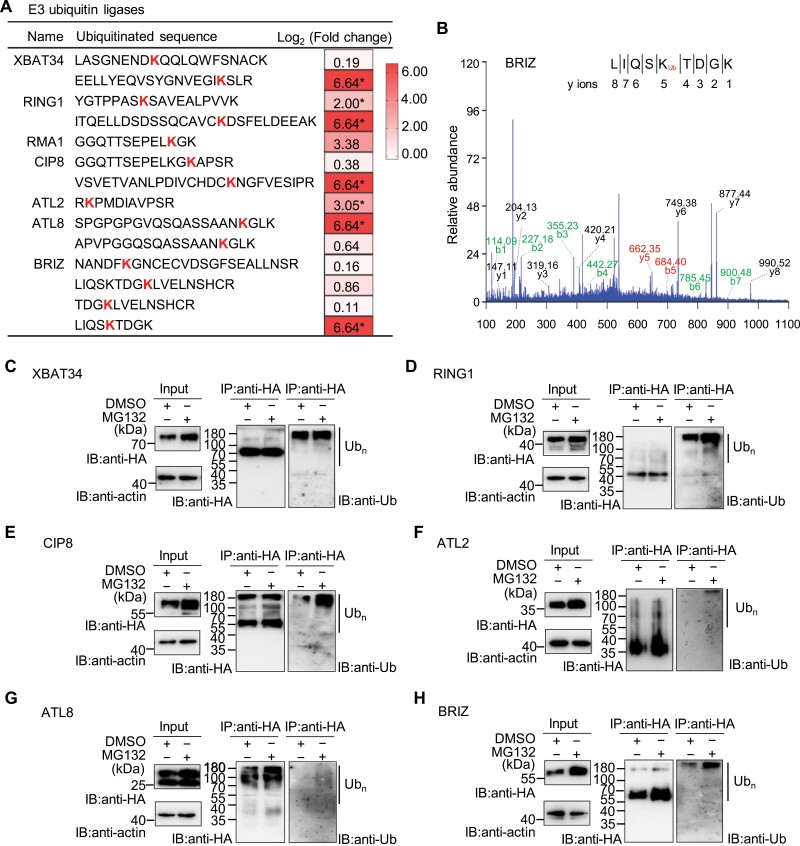
Identification of ubiquitination sites in E3 ubiquitin ligases and *in vivo* ubiquitination assay. (A) The list of E3 ubiquitin ligases containing ubiquitination sites. Heatmaps showed the log_2_-transformed fold changes of ubiquitination levels of the indicated ubiquitination sites after 100 μM MG132 treatment for 4 h. Asterisks indicate significant differences (fold change>2.0 or<0.5; *P* value<0.05, Student’s *t* test). ATL2, Arabidopsis Tóxicos en Levadura 2; ATL8, Arabidopsis Tóxicos en Levadura 8; BRIZ, BRAP2 RING ZnF UBP domain-containing protein; CIP8, COP1 interaction protein 8; RING1, PRC1 core component AtRING1; RMA1, RING membrane-anchor 1; XBAT34, XB3 Ortholog 3 in *Arabidopsis thaliana.* (B) Representative mass spectra of the ubiquitinated peptides for BRIZ. The y-ions and the corresponding peptide sequence are shown and the ubiquitinated lysine (K) residue is marked in red. (C–H) *In vivo* ubiquitination assay for E3 ubiquitin ligases listed in (A). The E3 ubiquitin ligases fused to the HA tag (E3s-HA) were transiently expressed in tobacco leaves followed by treatment with or without 100 μM MG132 for 4 h; DMSO=control. Total proteins extracted from tobacco leaves were immunoprecipitated with anti-HA antibody beads and subjected to immunoblot using either anti-HA or anti-ubiquitin antibody. Equal loading was confirmed by an anti-actin antibody. The poly-ubiquitin is indicated. IB, immunoblot; Ub, ubiquitin; (Ub)_n_, poly-ubiquitin chain.

E3 ubiquitin ligases primarily determine the substrate specificity (Callis, 2014). Out of the 25 E3s identified in our ubiquitinome, only seven E3s, namely XB3 Ortholog 3 in *Arabidopsis thaliana* (XBAT34), PRC1 core component AtRING1 (RING1), RING membrane-anchor 1 (RMA), COP1 interaction protein 8 (CIP8), *Arabidopsis* Tóxicos en Levadura 2 (ATL2), *Arabidopsis* Tóxicos en Levadura 8 (ATL8), and BRAP2 RING ZnF UBP domain-containing protein (BRIZ), contained significantly up-regulated ubiquitination levels at specific ubiquitination sites (*P*<0.05, Student’s *t* test) after MG132 treatment (Fig. 5A, B; Supplementary Table S6). Interestingly, all of them belong to RING-type families E3 ubiquitin ligases, which contain RING-finger domains and function as a single subunit or in multi-subunit complexes (Callis, 2014). The homologs for several of these E3s have been reported to participate in important biological processes. For example, CIP8 functions as an essential component of the COP1 light signalling complex responsible for ELONGATED HYPOCOTYL 5 (HY5) stability (Hardtke *et al.*, 2002), while the membrane-localized ATL2 and ATL8 were likely involved in early elicitor responses in plant defence signalling and in sugar starvation response, respectively (Serrano and Guzmán, 2004; Luo *et al.*, 2019). AtBRIZ was reported to be required for germination and early seedling development (Hsia and Callis, 2010).

To test whether these seven E3s were indeed ubiquitinated in response to MG132, the HA-tagged recombinant E3s were transiently-expressed in tobacco leaves and subjected to *in vivo* ubiquitination assays. Immunoblot analysis revealed that, except for RMA that was not successfully expressed, the rest of the E3 ligases (XBAT34, RING1, CIP8, ATL2, ATL8, and BRIZ) accumulated after treatment with MG132 (Fig. 5C-H, left panels). Most of them exhibited obviously increased abundances of poly-ubiquitin (poly-Ub) linked conjugates after MG132 treatment (Fig. 5C-H, right panels). Since poly-ubiquitination was implicated in protein proteolysis through the proteasome, we carried out *in vitro* degradation assays for these six E3s in the presence or absence of MG132. We found that only ATL8 showed no obvious changes in protein abundance after MG132 treatment (Supplementary Fig. S6), consistent with it being a membrane protein that was degraded through a non-UPS pathway (MacGurn *et al.*, 2012). In contrast, five E3 ligases (XBAT34, RING1, CIP8, ATL2, and BRIZ) appeared to be more stable when the proteasome was inhibited (Supplementary Fig. S6). Collectively, these data pointed out that five E3 ligases (XBAT34, RING1, CIP8, ATL2, and BRIZ) may undergo degradation via the UPS pathway.

### E3 ubiquitin ligase FaBRIZ is involved in strawberry fruit ripening

To examine whether the five RING-type E3 ubiquitin ligases (XBAT34, RING1, CIP8, ATL2, and BRIZ) described above participate in the regulation of strawberry fruit ripening, an RNA interference (RNAi) experiment was performed. The specific cDNA fragments of the five E3 genes were cloned and inserted into the RNAi vector, individually. The octoploid cultivated strawberry fruit at the large green (LG) stage was used for agroinfiltration and visual inspection. The results showed that fruits suppressed for *FaBRIZ* exhibited an obvious phenotype (Supplementary Fig. S7). The colour change in *FaBRIZ*-RNAi fruits happened earlier than in the controls after agroinfiltration (Supplementary Fig. S7), suggesting that FaBRIZ negatively regulates strawberry fruit ripening.

**Fig. 6.**
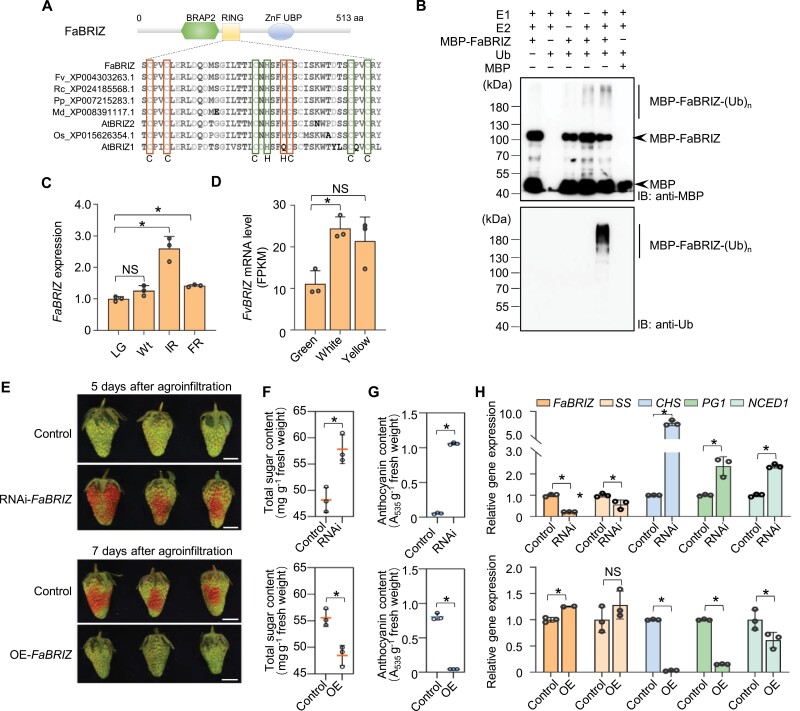
FaBRIZ functions as an E3 ubiquitin ligase to negatively regulate strawberry fruit ripening. (A) Characterization of the RING-type zinc finger domain (RING) in FaBRIZ. Sequence alignment of the RING domain shows the conserved amino acids. Protein domains are predicted using the SMART tool (http://smart.embl.de/). The BRCA-1 binding protein domain (BRAP2), RING domain, and zinc-finger ubiquitin binding domain (ZnF-UBP) are shown as hexagon, rectangle, and circle, respectively. C, cysteine; H, histidine. Fv, *Fragaria vesca*; Fa, *Fragaria×ananassa*; Rc, *Ricinus communis*; Pp, *Prunus persica*; Md, *Malus domestica*; Os, *Oryza sativa*; At, *Arabidopsis thaliana*. (B) *In vitro* ubiquitination assay of FaBRIZ. The ubiquitination reaction was conducted in the presence (+) or absence (–) of E1, E2, Ubiquitin (Ub), and MBP-FaBRIZ. The reaction products were detected by western blotting using anti-MBP (upper panel) and anti-ubiquitin (lower panel) antibodies. MBP protein was used as the negative control. (Ub)_n_, poly-ubiquitin chain; IB, immunoblot. (C) Expression of *FaBRIZ* in octoploid strawberry fruits at large green (LG), white (Wt), initial red (IR), and full red (FR) stages determined by quantitative RT–PCR. (D) Transcript levels of *FvBRIZ* in diploid woodland strawberry at different developmental stages (Green, White, and Yellow) revealed by RNA-seq (Zhou *et al.*, 2021). (E) Ripening phenotypes of *FaBRIZ* RNA interference (RNAi-*FaBRIZ*) and overexpression (OE-*FaBRIZ*) fruits. Strawberry fruits agroinfiltrated with empty vectors were used as controls. The experiments were performed with three biological replicates and each replicate contained 20 fruits. The representative results of RNAi-*FaBRIZ* (5 d after agroinfiltration) and OE-*FaBRIZ* (7 d after agroinfiltration) are presented. Scale bar=1 cm. (F) Detection of total sugar and (G) anthocyanin content in the RNAi- (upper panel) and OE- (lower panel) fruits. (H) Transcript levels of *FaBRIZ*, *sucrose synthase* (*SS*), *chalcone synthase* (*CHS*), *polygalacturonase 1* (*PG1*) and *9-cis-epoxycarotenoid dioxygenase 1* (*NCED1*), in the RNAi- (upper panel) and OE- (lower panel) fruits determined by quantitative RT–PCR. In (C), (H), the relative quantification was based on the cycle threshold (Ct) ^2(-ΔΔ Ct)^ method. The *Actin* gene was used as an internal control. The expression level of *FaBRIZ* at LG stage (C) and the ripening-related genes in control fruits (H) were defined as 1. Data are presented as means ±SD (*n*=3). Asterisks indicate significant differences (*P<*0.05, Student’s *t* test). The circles on the bars indicate the values of each independent experiment.

FaBRIZ is composed of 513 amino acids, containing a RING-type zinc finger domain (RING), a BRCA-1 binding protein domain (BRAP2), and a zinc-finger ubiquitin binding domain (ZF-UBP) ( Fig. 6A). Based on an alignment of homologous protein sequences, the RING domain in FaBRIZ is a C3H2C3-type zinc finger, with all eight predicted zinc-binding residues (Fig. 6A). To determine whether FaBRIZ functions as an E3 ligase, the MBP-tagged recombinant FaBRIZ protein (MBP-FaBRIZ) prepared from *Escherichia coli* was incubated with wheat E1, human E2, and Arabidopsis ubiquitin for an *in vitro* ubiquitination assay. Immunoblot analysis using anti-MBP and anti-ubiquitin antibodies showed that the signals of high molecular mass bands, which represent ubiquitinated FaBRIZ, were increased in the intact reaction system, compared with other reactions in the absence of a single component (Fig. 6B). These data indicated that FaBRIZ has E3 ubiquitin ligase activity *in vitro* and can catalyse its self-ubiquitination.

*FaBRIZ* gene in the octoploid cultivated strawberry exhibited increased expression from the white stage to the red stage (Fig. 6C; Supplementary Fig. S8A). Expression of the homologous gene of *FaBRIZ* in wild strawberry (*FvBRIZ*) also increased significantly from green stage to white stage based on previous transcriptome data (Zhou *et al.*, 2021) (Fig. 6D). These data confirmed that FaBRIZ may play a role in the regulation of strawberry fruit ripening. To further verify the function of FaBRIZ in regulating fruit ripening, we generate an overexpression (OE) construct of *FaBRIZ* under the control of a 35S cauliflower mosaic virus promoter and then transformed them into the octoploid strawberry fruit. As expected, we found that overexpression of *FaBRIZ* delayed fruit ripening (Fig. 6E). A visible colour change was observed at 7 d after agroinfiltration in the control, whereas OE-*FaBRIZ* fruits were almost green at this stage (Fig. 6E). Fruit quality analysis indicated that total sugar and anthocyanin content were higher in the RNAi fruits (upper panel) and lower in the OE fruits (lower panel; Fig. 6F, G). Gene expression analysis of RNAi and OE fruits indicated that *FaBRIZ* was successfully silenced in the RNAi fruits while enhanced in the OE fruits (Fig. 6H; Supplementary Fig. S8B). The expression of the ripening genes *chalcone synthase* (*CHS*), *polygalacturonase 1* (*PG1*), and *9-cis-epoxycarotenoid dioxygenase 1* (*NCED1*) was dramatically enhanced in the *FaBRIZ* RNAi-fruits, but displayed a noticeable decrease in the OE fruits (Fig. 6H; Supplementary Fig. S8B). These results suggest that FaBRIZ is necessary for normal fruit ripening of strawberry. We detected the ubiquitination changes of total proteins in *FaBRIZ* RNAi and OE fruits and found that high-molecular weight polyubiquitinated proteins increased in RNAi fruits but decreased in OE fruits (Supplementary Fig. S8C). This suggests that FaBRIZ might directly or indirectly affect ubiquitination of total proteins. FaBRIZ may therefore function as a negative regulator through UPS-mediated regulation of certain positive factors in the process of fruit ripening in strawberry.

## Discussion

The UPS has been shown to regulate multiple ripening proteins, including proteins related to hormone synthesis and signalling (Yang *et al.*, 2010; Shan *et al.*, 2020; Yu *et al.*, 2021; Song *et al.*, 2022), transcription factors (Hu *et al.*, 2019; Yang *et al.*, 2022), and key enzymes associated with fruit quality (Tang *et al*, 2016; Wang *et al.*, 2020; Wei *et al.*, 2021). However, global identification of substrates that are targeted by UPS in fruits is still lacking. Here, we found that the ripening of strawberry fruits was delayed by the proteasome inhibitor MG132. We then carried out a full ubiquitinome analysis of strawberry fruits using nano-HPLC-MS/MS combined with K-ɛ-GG peptide immunoprecipitation. A total of 2947 ubiquitination sites for 2878 ubiquitinated peptides corresponding to 1487 proteins were successfully identified, confirming this method as being a powerful approach in mapping ubiquitinated sites. Our result is comparative to previous ubiquitinome data in other plants which identified 1500–2500 ubiquitination sites (Guo *et al.*, 2017; Wang *et al.*, 2019; Gu *et al.*, 2019).

### Ubiquitin-proteasome system regulates strawberry fruit ripening by targeting ripening-related proteins

Fruit ripening depends upon the expression of multiple ripening-related genes, which encode key enzymes to catalyse a range of biochemical changes (Li *et al.*, 2021). In this study, we identified ubiquitination sites in crucial enzymes in sugar and acid metabolism, such as SS, SPP, MDH1, and IDH1, in cell wall modification, such as CSLC4, MPE3, and CESA, in anthocyanin synthesis, such as CHS, CHI, and ANR, and in ABA biosynthesis, such as NCED1 (Fig. 4; Supplementary Table S5). Some of them exhibited altered ubiquitination levels when the proteasome was inhibited. Ubiquitination has been reported to regulate anthocyanin biosynthesis by mediating degradation of anthocyanin biosynthesis-related enzymes and transcription factors, such as phenylalanine ammonialyase (PAL), CHS, and MYB1 transcription factor (Zhang *et al.*, 2013; Wang *et al.*, 2018; Gu *et al.*, 2019). ABA plays important roles in the regulation of strawberry fruit ripening. It is reported that ABA levels in the receptacles of strawberry fruit gradually increased during the whole fruit development and ripening (Jia *et al.*, 2011). The UPS-mediated degradation of key proteins, such as NCED1, PYL8/9, PP2C, and ABI5 (Lee *et al.*, 2010; Irigoyen *et al.*, 2014; Kong *et al.*, 2015), has been demonstrated to be a critical regulatory mechanism in the ABA biosynthesis and signalling pathway. Moreover, the homologs for some of the enzymes in sugar and acid metabolism have been reported to be ubiquitinated and degraded by the ubiquitin-proteasome pathway in plants, yeast, and humans, such as sucrose synthase (SUS; Hardin and Huber, 2004), enolase 1 (ENO1; Zhan *et al.*, 2015), IDH1 (Xu *et al.*, 2019), MDH1 and fructose-1,6-bisphosphatase (FBP; Hung *et al.*, 2004).

The UPS might be activated to dynamically control the turnover of key ripening-related enzymes, preventing their over-accumulation in fruits. Compared with changes in transcription, the alterations at protein levels regulated by ubiquitination may represent a fast and efficient way to regulate gene expression. Once the ripening program is started, the ripening-related enzymes are rapidly accumulated, concomitant with the activation of UPS to maintain protein homeostasis. Indeed, some ripening-related enzymes were present along with the corresponding E3 ligases at the same stage of fruit ripening, making it available for their potential interactions and quantity control (Hu *et al.*, 2019; Wang *et al.*, 2020; Ling *et al.*, 2021). Our results highlight the importance of homeostasis of enzymes involved in carbohydrate metabolism, cell wall synthesis, anthocyanin biosynthesis, and ABA biosynthesis in the early stages of fruit ripening, and imply the potential roles of UPS in controlling this process.

Our ubiquitinome also identified some other proteins that were well reported to be associated with fruit ripening. For instance, a number of ripening-related transporters appeared in our list of ubiquitinated proteins (Supplementary Table S5), such as aluminium-activated malate transporter 4 (ALMT4), a major malate transporter essential for malate accumulation (Hu *et al.*, 2017), early response to dehydration like 6 (ERD6), a tonoplast H^+^/glucose symporter involved in sugar accumulation ( Zhu *et al.*, 2021), and DETOXIFICATION 41 (DTX41), the ortholog of Arabidopsis AtTT12 and grape VcMATE2 that participate in vacuolar transportation of proanthocyanidins and anthocyanin, respectively (Marinova *et al*, 2007; Pérez-Díaz *et al.*, 2014), implying a key role for ubiquitination in controlling transporter turnover or trafficking. Within these transporter proteins, the ubiquitination level for specific ubiquitination sites in DTX41 was up-regulated after MG132 treatment (Supplementary Table S6), indicating that the ubiquitin-proteasome pathway is capable of regulating the levels of this protein. Interestingly, we identified ubiquitination sites on key enzymes in the methionine cycle pathway, such as S-adenosylmethionine synthase (SAMS), S-adenosyl-L-homocysteine hydrolase (SAHH), and various S-adenosyl-L-methionine dependent methyltransferases (MET) (Supplementary Table S5). These proteins are needed to recycle S-adenosylmethionine homocysteine (SAH) to S-adenosylmethionine (SAM), whose methyl group will be transferred to different target biomolecules for methylation, such as DNA, RNA, and histone (Watanabe *et al.*, 2021). It is known that DNA and RNA methylation play important roles in the regulation of fruit ripening (Lang *et al.*, 2017; Zhou *et al.*, 2021), and therefore it would be interesting to further study whether the methylation processes are influenced by the ubiquitin-proteasome pathway during fruit ripening.

We did not identify any ubiquitination sites for those well-known ripening-related transcription factors, such as SHATTERPROOF-like (FaSHP; Daminato *et al.*, 2013), Tomato MADS box gene6 (FaTM6; Martín-Pizarro *et al.*, 2019) and Ripening Inducing Factor (FaRIF; Martín-Pizarro *et al.*, 2021) in strawberry. It is possible that the ripening stage of strawberry fruit used in this study was at an early stage, when those proteins are present at very low abundance. However, our ubiquitinome analysis identified 35 ubiquitination sites within 27 putative transcription factors, including the members of the MADS-box family (FvMADS2), the NAC family (FvNAC29), the ERF family (RAP2), and the MYB family (MYB44) (Supplementary Table S5). Whether these transcription factors are necessary for strawberry fruit ripening requires further investigation.

### Ubiquitin-proteasome system regulates strawberry fruit ripening by targeting its own components

Multiple UPS components have been identified in previous ubiquitinome studies in Arabidopsis, rice, and other plants (Kim *et al.*, 2013; Guo *et al.*, 2017; Wang *et al.*, 2019). Similar to these results, our study of the ubiquitinome in strawberry fruit also identified a large number of UPS-related proteins covering proteasome subunits, ubiquitin activating enzyme E1, ubiquitin conjugating enzymes E2s, ubiquitin ligases E3s, deubiquitinated enzymes, and ubiquitin receptors (Supplementary Table S6). The original explanation for the identification of these UPS-related proteins is that they were not direct targets of ubiquitination but by-products during UPS-mediated proteolysis (Kim *et al.*, 2013). However, it was later reported that UPS components (e. g. PSMD4/Rpn10, PSMC3/Rpt5, and Uch37) were dynamically modified by ubiquitination and de-ubiquitination, which can function to adjust proteasomal activity in processing ubiquitinated proteins (Jacobson *et al.*, 2014).

E3 ubiquitin ligases directly modulate ubiquitination levels and determine the specificity of the targeted substrates. A number of E3 ubiquitin ligases along with their targets have been reported to function in the ripening process of various fruits, such as tomato (Yang *et al.*, 2010; Tang *et al.*, 2016; Wang *et al.*, 2020; Ling *et al.*, 2021), banana (Shan *et al.*, 2020; Song *et al.*, 2022; Yang *et al.,* 2022), apple (Shan *et al.*, 2020; Wei *et al.*, 2021), and grape (Yu *et al.*, 2021). In this study, by combining diGly-ubiquitinome analysis, ubiquitination assay, and RNAi experiments, we identified a strawberry E3 ligase FaBRIZ, which functions as a negative regulator in fruit ripening. Our study not only represents a strategy for discovering new E3 ligases in certain biological processes, but also provides insights into the role of E3 ligases in controlling strawberry fruit ripening. Recently, it was reported that the homolog of FaBRIZ in Arabidopsis, AtBRIZ2, was capable of combining with AtBRIZ1, a close ortholog of AtBRIZ2, to form a functional ubiquitin E3 ligase complex, and regulate seed germination and seeding growth (Hsia and Callis, 2010; Linden *et al.*, 2021). This modulation might be achieved through the regulation of ABA signalling and response by these two BRIZ proteins (Linden *et al.*, 2021). Considering that ABA plays a key role in the regulation of strawberry fruit ripening (Jia *et al.*, 2011), it is conceivable that FaBRIZ might regulate fruit ripening through directly or indirectly affecting the ABA pathway, although the direct targets of FaBRIZ remain to be determined.

In conclusion, by using a state-of-the-art technique for comprehensive identification of ubiquitinated proteins and the corresponding ubiquitination sites, we reveal that a number of ripening-related proteins in strawberry fruit contain ubiquitination sites and may be regulated by UPS-mediated protein degradation. We further identified FaBRIZ as a novel RING-type E3 ligase that functions as a negative regulator for strawberry fruit ripening. Our ubiquitinome data provide a basis for further investigation of UPS-mediated regulation of fruit ripening.

## Supplementary data

The following supplementary data are available at *JXB* online.

Fig. S1. Determination of ubiquitination of total proteins in strawberry fruits after proteasome inhibition.

Fig. S2. Ripening phenotypes of strawberry fruits after proteasome inhibition.

Fig. S3. Gene Ontology (GO) analysis of ubiquitinated proteins.

Fig. S4. Quantitative proteomic profiling of strawberry fruits after proteasome inhibition.

Fig. S5. KEGG enrichment analysis of ubiquitinated proteins with differentially-regulated ubiquitination sites after proteasome inhibition.

Fig. S6. Cell-free degradation assays for E3 ubiquitin ligases.

Fig. S7. Specific E3 ubiquitin ligases are involved in the regulation of strawberry fruit ripening.

Fig. S8. Ripening gene expression and total protein ubiquitination in *FaBRIZ* RNAi- and OE-fruits.

Table S1. PCR primers sequences used in this study.

Table S2. List of all identified ubiquitination sites in strawberry fruits.

Table S3. List of significantly changed ubiquitination sites in strawberry fruits after proteasome inhibition.

Table S4. List of all identified proteins in strawberry fruits by TMT-labelling proteomic analysis.

Table S5. Ripening-related proteins containing ubiquitination sites in strawberry fruits.

Table S6. Components of the ubiquitin-proteasome system containing ubiquitination sites in strawberry fruits.

erac400_suppl_Supplementary_Figures_S1-S8

erac400_suppl_Supplementary_Table_S1

erac400_suppl_Supplementary_Table_S2

erac400_suppl_Supplementary_Table_S3

erac400_suppl_Supplementary_Table_S4

erac400_suppl_Supplementary_Table_S5

erac400_suppl_Supplementary_Table_S6

## Data Availability

Mass spectrometry data for the ubiquitinated peptide identification and the TMT-based quantitative proteomics have been deposited in the OMIX (https://ngdc.cncb.ac.cn/omix/) in National Genomics Data Center (NGDC) under the accession number OMIX001260 and OMIX001261. Other data supporting the findings of this study are available in the paper and its supplementary data.
