# Supplementary material for: Global ubiquitinome analysis reveals the role of E3 ubiquitin ligase FaBRIZ in strawberry fruit ripening
Source: J Exp Bot. 2022 Oct 10;74(1):214–32. doi: 10.1093/jxb/erac400 (PMC9786855; doi:10.1093/jxb/erac400)
Supplement: erac400_suppl_Supplementary_Figures_S1-S8 [file erac400_suppl_supplementary_figures_s1-s8.pdf]

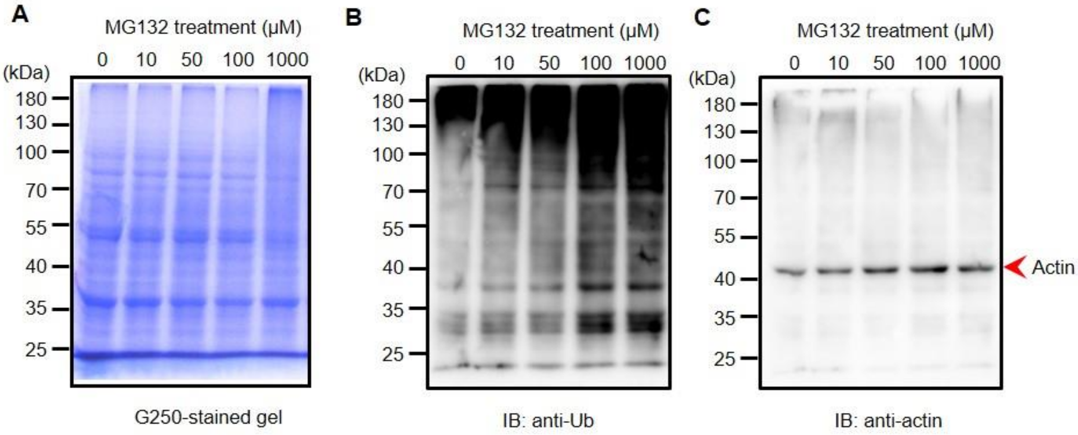

Fig. S1 Determination of ubiquitination of total proteins in strawberry fruits after proteasome inhibition. Proteins extracted from strawberry fruits at large green (LG) stage after treatment with different concentrations of MG132 for 4 hours were separated by 10% SDS-PAGE and subjected to western blot analysis. (A) Gels stained with Coomassie Blue (G250). (B, C) Immunoblot (IB) detection of ubiquitinated proteins by anti-ubiquitin antibody (P4D1) (B), and actin by an anti-actin antibody (C). Arrow indicates actin signal.

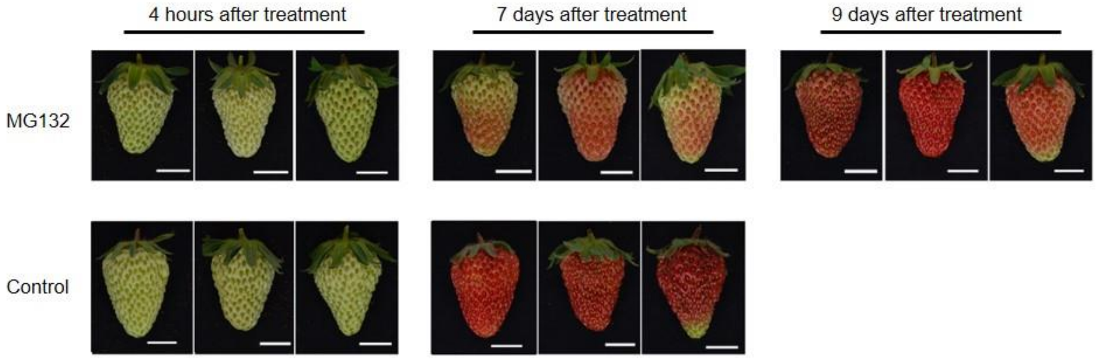

Fig. S2 Ripening phenotypes of strawberry fruits after proteasome inhibition. Strawberry fruits at large green (LG) stage were injected with 100 μM of MG132 or DMSO solution (control). The experiments were performed with more than three biological replicates and each replicate contained 20 fruits. The representative photographs of fruits at 4 hours, 7 days, and 9 days, were shown. Scale bar = 1 cm.

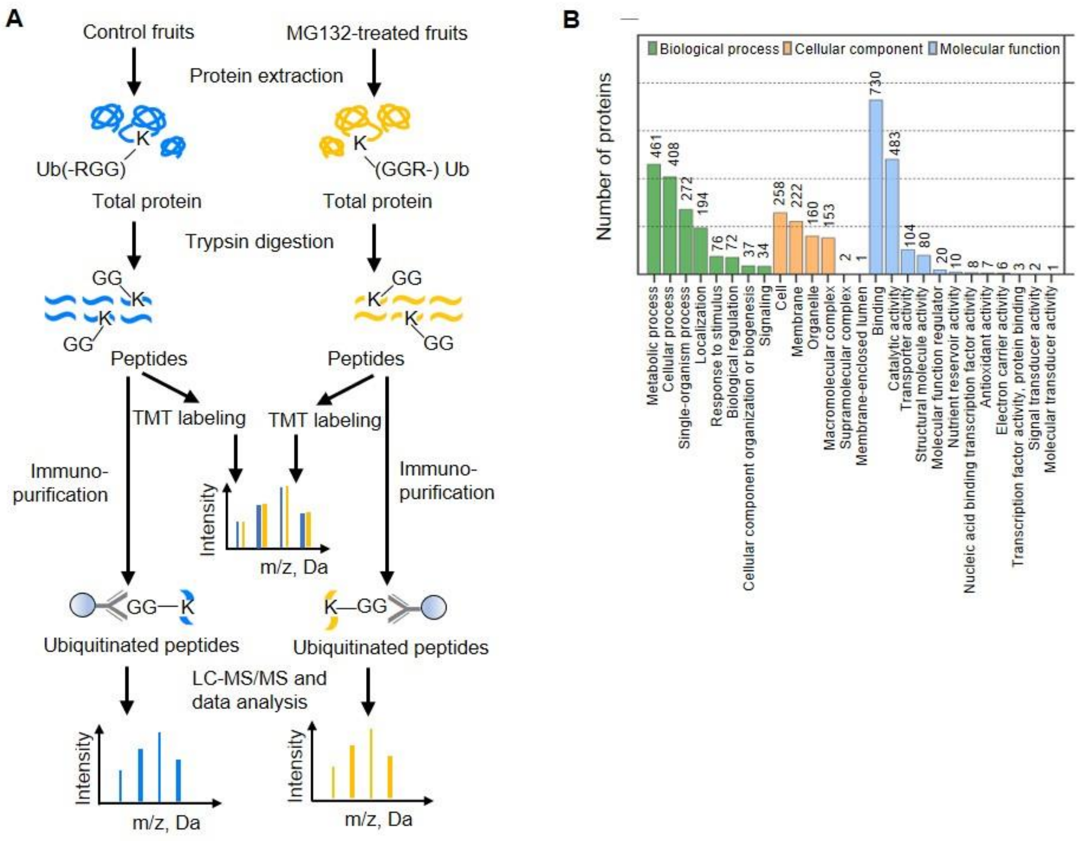

Fig. S3 Gene Ontology (GO) analysis of ubiquitinated proteins. (A) Workflow of ubiquitinome analysis of strawberry fruits after 4 hours of MG132 treatment. The proteins were extracted and digested overnight with trypsin. Following anti-K-ε-GG antibody enrichment, the ubiquitinated peptides were analyzed by nano-HPLC-MS/MS using label-free quantification method. (B) Gene Ontology (GO) analysis of total ubiquitinated proteins identified in strawberry fruits treated with or without MG132. The number of proteins belonging to each GO category is shown.

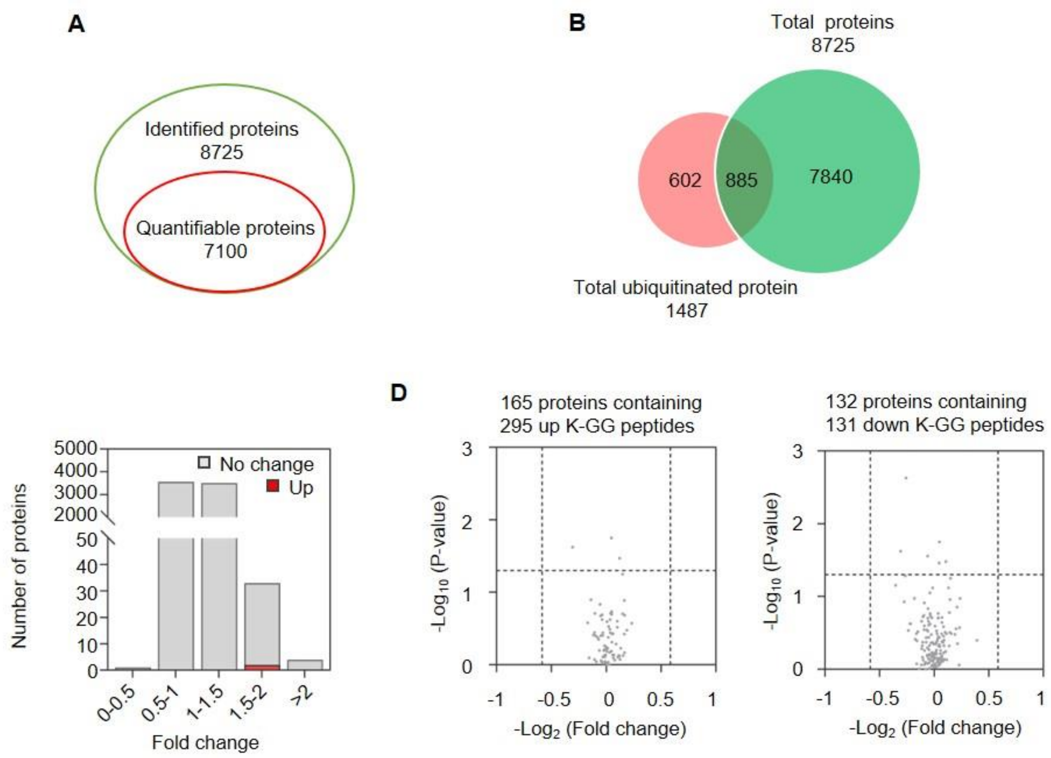

Fig. S4 Quantitative proteomic profiling of strawberry fruits after proteasome inhibition. (A) Number of identified and quantified proteins. (B) Ven diagrams showing the numbers of overlapping proteins identified in ubiquitinome and proteome, respectively. (C) Fold change distribution of differently regulated proteins. Fold change > 1.5 or < 0.67 ($P < 0.05$) cut-off were considered statistically significant. (D) Volcano plots showing the log_2-transformed fold changes of proteins containing differentially-regulated ubiquitination sites (fold change > 2.0 or < 0.5, P value < 0.05). Proteins with significantly higher and lower abundance are highlighted in red and green, respectively. Proteins with no significant change in abundance are colored in grey.

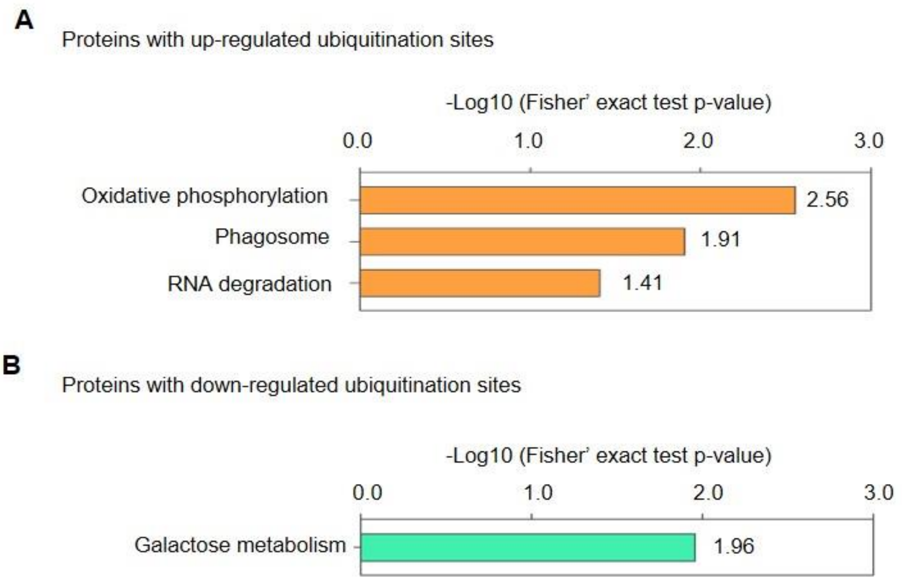

Fig. S5 KEGG enrichment analysis of ubiquitinated proteins with differentially-regulated ubiquitination sites after proteasome inhibition. Proteins with up-regulated (A) and down-regulated (B) ubiquitination sites after MG132 treatment were used for analysis, respectively. The q value of pathway enrichment determined by Fisher's exact test with Bonferroni correction is shown.

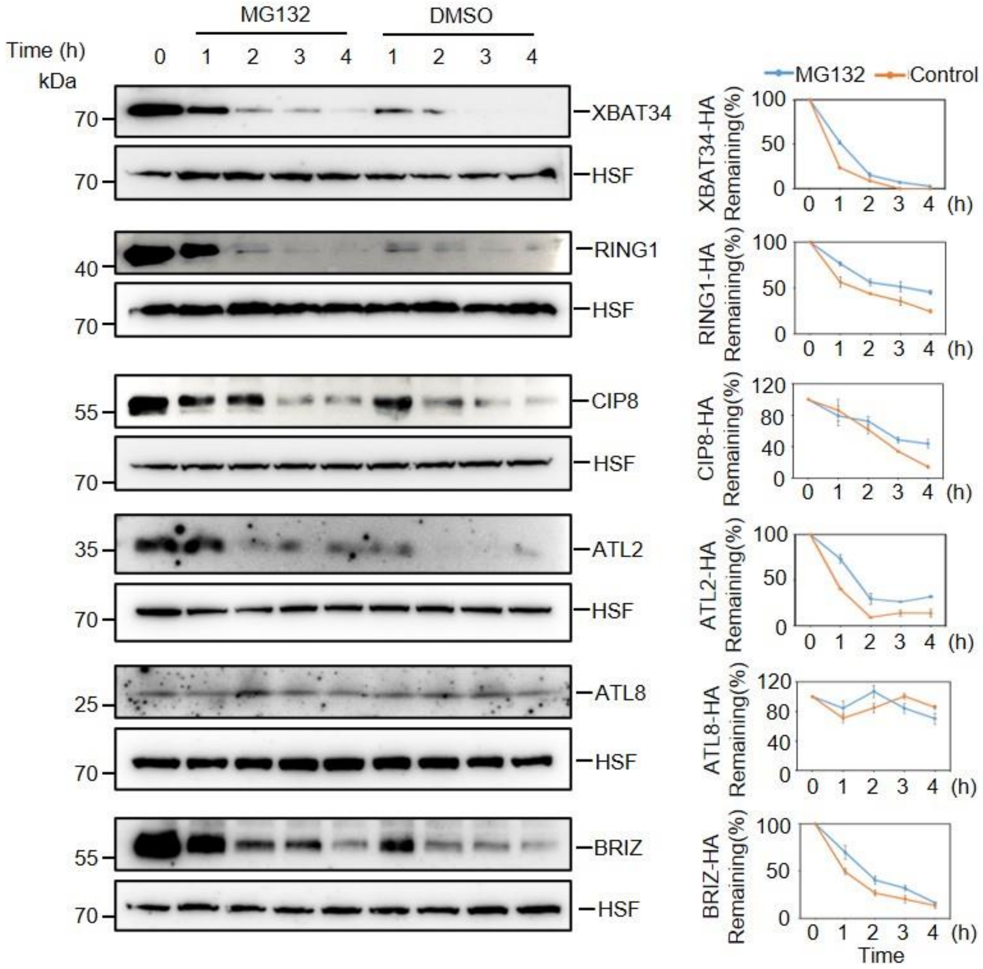

Fig. S6 Cell-free degradation assays for E3 ubiquitin ligases. The E3 ligases fused to the HA tag (E3s-HA) were transiently expressed in tobacco leaves. Proteins extracted from leaves were incubated with or without MG132 for *in vitro* degradation assays at different time intervals. Protein levels were determined by immunoblot using anti-HA antibody and quantification of protein levels by ImageJ is shown. Data are presented as means ± standard deviations (n = 3). XBAT34, XB3 Ortholog 3 in *Arabidopsis thaliana*; RING1, PRC1 core component AtRING1; CIP8, COP1 interaction protein 8; ATL2, *Arabidopsis* Tóxicos en Levadura 2; ATL8, *Arabidopsis* Tóxicos en Levadura 8; BRIZ, BRAP2 RING ZnF UBP domain-containing protein.

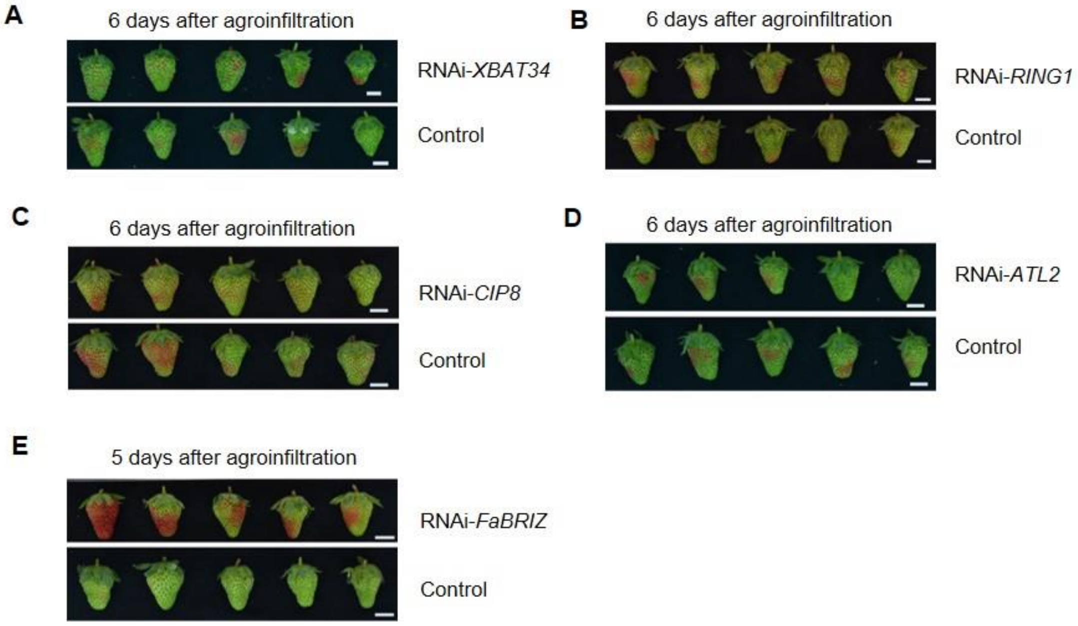

Fig. S7 Specific E3 ubiquitin ligases are involved in the regulation of strawberry fruit ripening. Ripening phenotypes of XBAT34- (A), RING1- (B), CIP8- (C), ATL2- (D), and *FaBRIZ*-RNA interference (RNAi) fruits (E). Strawberry fruits agroinfiltrated with empty plasmids were used as controls. The experiments were performed with three biological replicates and the representative results are presented. Scale bar = 1 cm.

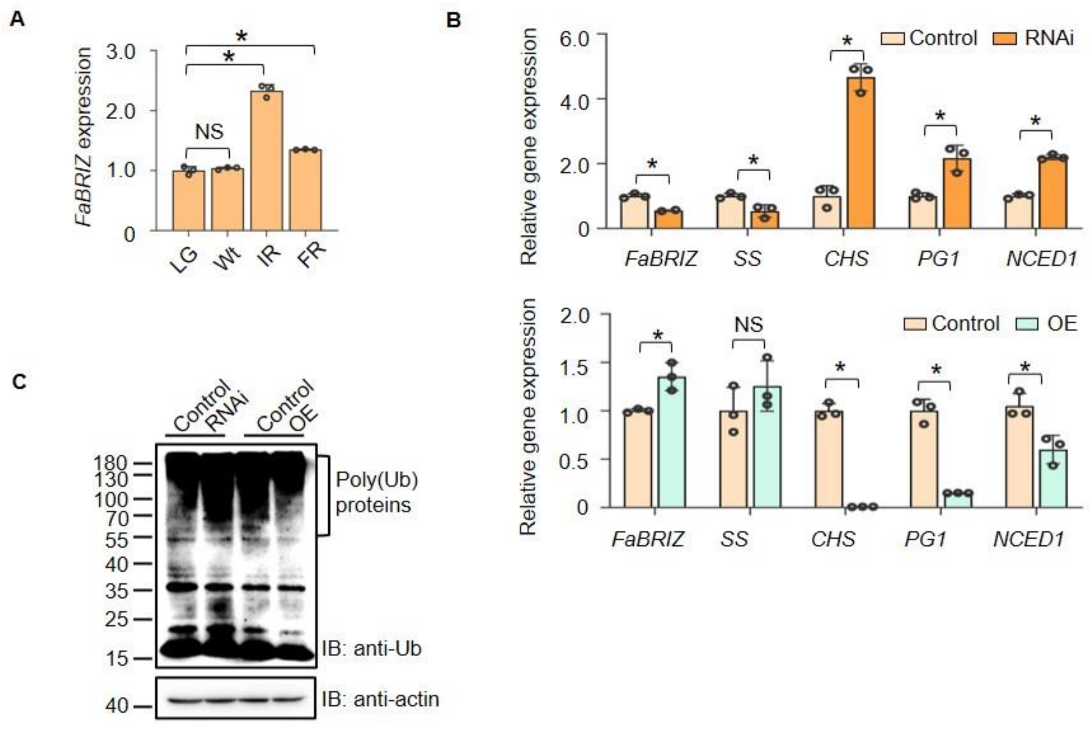

Fig. S8 Ripening gene expression and total protein ubiquitination in *FaBRIZ* RNAi- and OE-fruits. (A) Expression of *FaBRIZ* in octoploid strawberry fruit at large green (LG), white (Wt), initial red (IR), and full red (FR) stage determined by quantitative RT-PCR. (B) Expression of *FaBRIZ*, *sucrose synthase* (*SS*), *chalcone synthase (CHS)*, *polygalacturonase 1 (PG1)*, and *9-cis-epoxycarotenoid dioxygenase 1* (*NCED1*), in the RNAi- (upper panel) and OE- (lower panel) fruits determined by quantitative RT-PCR. In (A)-(B), the *GAPDH* gene was used as an internal control. Data are presented as means ± standard deviations (n = 3). Asterisks indicate significant differences ($P < 0.05$, Student's *t* test). (C) Immunoblot (IB) detection of ubiquitinated proteins by anti-ubiquitin antibody (P4D1). Protein extracts were prepared from RNAi- or OE-fruits and the control. Actin served as the loading control.
